# Genomics‐assisted breeding for designing salinity‐smart future crops

**DOI:** 10.1111/pbi.70104

**Published:** 2025-05-20

**Authors:** Ali Raza, Qamar U. Zaman, Sergey Shabala, Mark Tester, Rana Munns, Zhangli Hu, Rajeev K. Varshney

**Affiliations:** ^1^ Guangdong Key Laboratory of Plant Epigenetics, College of Life Sciences and Oceanography Shenzhen University Shenzhen China; ^2^ Shenzhen Engineering Laboratory for Marine Algal Biotechnology, Guangdong Technology Research Center for Marine Algal Biotechnology, Longhua Innovation Institute for Biotechnology, College of Life Sciences and Oceanography Shenzhen University Shenzhen China; ^3^ School of Breeding and Multiplication, Hainan Yazhou Bay Seed Laboratory Hainan University Sanya China; ^4^ School of Biological Sciences The University of Western Australia Perth WA Australia; ^5^ International Research Centre for Environmental Membrane Biology Foshan University Foshan China; ^6^ Center of Excellence for Sustainable Food Security and Division of Biological and Environmental Sciences and Engineering King Abdullah University of Science and Technology (KAUST) Thuwal Saudi Arabia; ^7^ Centre of Excellence in Plant Energy Biology, School of Molecular Sciences The University of Western Australia Perth WA Australia; ^8^ Guangdong Provincial Key Laboratory of Functional Substances in Medicinal Resources and Healthcare Products, School of Life Sciences and Food Engineering Hanshan Normal University Chaozhou China; ^9^ WA State Agricultural Biotechnology Centre, Centre for Crop and Food Innovation, Food Futures Institute Murdoch University Murdoch WA Australia

**Keywords:** cell‐/tissue‐based phenotyping, crop wild relatives, genome sequencing, pan‐genomics, salinity tolerance, single‐cell genomics

## Abstract

Climate change induces many abiotic stresses, including soil salinity, significantly challenging global agriculture. Salinity stress tolerance (SST) is a complex trait, both physiologically and genetically, and is conferred at various levels of plant functional organization. As both the sustainability and profitability of agricultural production systems are critically dependent on SST, plant breeders are trying to design and develop salinity‐smart crop plants capable of thriving under high salinity conditions. The accessibility of extreme‐quality reference genomes for cultivated crops, naturally salinity‐smart plants, and crop wild relatives has fast‐tracked the discovery of key genes and quantitative trait loci (QTLs), marker development, genotyping assays and molecular breeding products with improved SST. Employing fast‐forward breeding tools, namely genomic selection (GS), haplotype‐based breeding (HBB), artificial intelligence (AI) and high‐throughput phenotyping (HTP), has shown influence not only for fast‐tracking genetic gains but also for reducing the time and cost of developing commercial cultivars with enhanced SST and yield stability. This review discusses the advancement and prospects of various genomics‐assisted breeding (GAB) tools, including genome sequencing, QTL mapping, GWAS, GS, HBB, pan‐genomics, single‐cell/tissue genomics and phenotyping, epigenomics and transgenomics, to exploit the genetic landscape for improving SST. Additionally, we explore the integration of HTP and AI, which demonstrates how these innovative approaches can optimize breeding efficiency and guide large‐scale breeding efforts for designing salinity‐smart crops to ensure sustainable agriculture and global food security. The collective adoption of these tools suggests bridging the gap between research and field application to deliver stress‐smart varieties designed for saline‐affected regions worldwide.

## Graphical abstract

This review explores fast‐forward genomics‐assisted breeding tools to fast‐track the designing of salinity‐smart crops. Integration of various breeding tools can enhance salinity tolerance, ensuring sustainable agriculture and global food security.

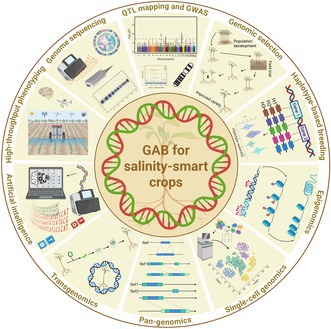

## Setting the stage: Why do we need to design salinity‐smart plants?

**Figure 1:**
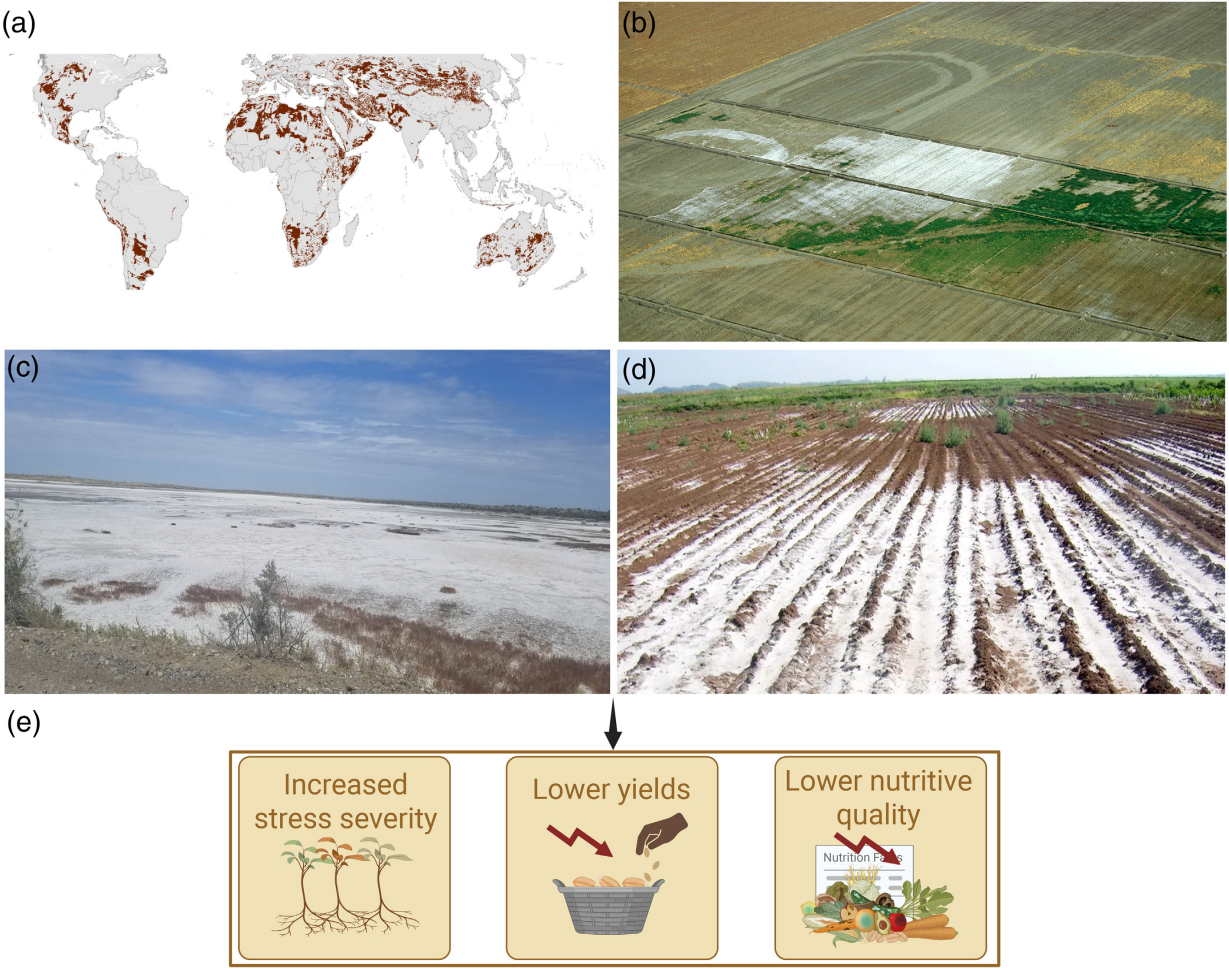
Impact of salinity stress on agriculture and food. (a) Map of all salt‐affected fields (>4 dS/m EC_e_), posing significant threats to crop production on a global scale. Adapted from Negacz *et al*. (2022) under CC BY 4.0 international licence. (b–d) Soil salinity is one of the major outcomes of climate change. These images show that agricultural land is rapidly lost due to high soil salinity levels. Photo b exhibits aerial observation of fields in central California (western San Joaquin Valley) experiencing high salinity (source: Agricultural Research Service, United States Department of Agriculture; https://www.ars.usda.gov/oc/images/photos/sep04/k4500‐12/). Photo c showcases a landscape covered with a crust of salinity and was captured in Bukhara region of Uzbekistan near the Uzbek‐Turkmen border (source: ‘Photo contest on salt‐affected soils’ by FAO; https://www.fao.org/events/global‐symposium‐on‐salt‐affected‐soils/photo‐contest/en. Photo d exhibits salinity‐affected lands in Ethiopia, highlighting the global scope of this challenge (source: https://www.biosaline.org/news/2018‐07‐17‐6571). (e) The major impacts of high salinity on agriculture comprise reduced biological yields, decreased nutritional quality, increased stress severity and decreased food security. The urgent need to address these challenges is evident, insisting on designing salinity‐smart crop plants to uphold global food systems.

Global food security is jeopardized by both climate change and population growth (see https://www.fao.org/documents/card/en/c/cb3673en for more details). It is anticipated that the global demand for food will rise by 50%, and yields may decrease by up to 30% by 2050 due to global climate change (Rivero *et al*., 2022; Zandalinas *et al*., 2021a, 2024). Consequently, climate change and the subsequent upsurge of extreme meteorological events have shifted plant breeders' priority for breeding climate‐smart crop plants (Raza *et al*., 2025a; Rivero *et al*., 2022). Climate change can impose multiple stresses, and these stresses alone or in combination harm crop yields, lower nutritive quality and cause challenges for future food security (Raza *et al*., 2025a, 2025b; Rivero *et al*., 2022; Zandalinas *et al*., 2021a, 2024). Of these, salt stress is a foremost threat to global agriculture, leading to the deprivation of arable lands, mainly in heavily irrigated areas (Figure 1) (Ashraf and Munns, 2022; Hassani *et al*., 2020; Melino and Tester, 2023; Negacz *et al*., 2022; Raza *et al*., 2023d).

Unsustainable farming activities and population pressure are causing soil degradation worldwide, affecting over 1125 mha of arable land in salt‐affected countries (Hossain, 2019; Liu *et al*., 2020). Recent estimates also suggest that >10% of cropland is already disturbed by salinity, posing a substantial risk to food security (https://www.fao.org/newsroom/detail/world‐soil‐day‐fao‐highlights‐threat‐of‐soil‐salinization‐to‐food‐security‐031221/en). Notably, soil salinity results in a projected annual loss of US$ 31 million in agricultural productivity, making up to 1.5 mha of cropland unproductive each year and reducing the production capacity of up to 46 mha annually (https://www.fao.org/global‐soil‐partnership/areas‐of‐work/soil‐salinity/en/). Moreover, salinity‐affected soils are widespread across continents and climatic zones, especially in arid and semi‐arid localities (see FAO news for more info: https://www.fao.org/newsroom/detail/world‐soil‐day‐fao‐highlights‐threat‐of‐soil‐salinization‐to‐food‐security‐031221/en). The most salt‐affected countries are China, Australia, Kazakhstan, United States, Argentina, Libya, Algeria, Iran, Iraq, Mongolia, Namibia, India, Pakistan and Egypt (Hopmans *et al*., 2021; Negacz *et al*., 2022).

Salinization and sodification are key processes of soil degradation, affecting agricultural productivity by hindering seed germination, plant growth, vigor and crop yields by imposing various constraints, including water and oxidative stress, nutritional imbalances, ion toxicity and disturbance to key cell metabolism (Kumar *et al*., 2022; Melino and Tester, 2023; Morton *et al*., 2019; Munns *et al*., 2020; Munns and Gilliham, 2015; Raza *et al*., 2023c, 2023d; Roy *et al*., 2014). The mechanistic basis of plants' responses and adaptation to salinity stress has been comprehensively reviewed in recent years (Ashraf and Munns, 2022; Flowers and Colmer, 2015; Kotula *et al*., 2024; Kumar *et al*., 2022; Maré *et al*., 2020; Melino and Tester, 2023; Morton *et al*., 2019; Munns *et al*., 2020; Munns and Gilliham, 2015; Negrão *et al*., 2017; Rawat *et al*., 2022; Raza *et al*., 2023c, 2023d; Roy *et al*., 2014; Shabala and Munns, 2017; Shelden and Munns, 2023; Venkataraman *et al*., 2021; Zhao *et al*., 2020). These previous reviews have extensively explored plant responses to salinity at different levels, along with various management strategies. Still, a critical gap remains in establishing the power of GAB tools and their integration with modern breeding methods. Therefore, addressing this gap is essential to accelerate the design of salinity‐smart crop plants through innovative research directions and targeted breeding strategies.

Considering the above argument, there is an urgent necessity to prioritize investigation efforts to enhance salinity stress tolerance (SST) and mitigate its adverse effects on agriculture during the increasing global salinization. Understanding the biological responses of plants to salinity stress is also vital, especially in field conditions where plants face multiple abiotic stresses. Moreover, it is also important to gain insights into how plants incorporate salinity stress with growth and developmental events to combat the threat of crop yield losses. The question is: how can this knowledge be incorporated into the breeding programs?

Breeding remains crucial for improving crop production, given the limitations of stress mitigation by salinity management options in salinity‐affected regions. Although conventional breeding methods have yielded considerable advancements in some crops, they are often time‐consuming and reliant on genetic variability (Acquaah, 2015; Gaba *et al*., 2021; Melino and Tester, 2023; Raza *et al*., 2023d). We need to employ fast‐forward breeding and adaptation strategies to design salinity‐smart crops. In this context, genomics‐assisted breeding (GAB) emerges as a promising complementarity to conventional methods, accelerating the production of climate‐smart cultivars via comprehensive assessment and targeted genetic interventions (Bohra *et al*., 2020; Raza *et al*., 2024b; Varshney *et al*., 2005, 2021c). Moreover, the integration of GAB techniques with high‐throughput phenotyping (HTP) and artificial intelligence (AI) enables the estimation of phenotype from genotype, accelerating the integration of desirable traits into salinity‐smart cultivars. GAB harnesses modern genome wealth to characterize allelic variation and advance germplasm. Nonetheless, maintaining progress depends on new approaches to effectively manipulate allelic diversity for designing future cultivars (Bohra *et al*., 2020; Raza *et al*., 2024b; Varshney *et al*., 2021c). Future breeding efforts need to focus on advancing crop genomes by collecting beneficial alleles to enhance SST food crops.

This review provides a comprehensive platform that goes beyond conventional discussions of plant responses to salinity stress. First, we summarize the detrimental effect of salinity stress on crop growth/yields and highlight the urgency for designing salinity‐smart future crops. Then, we present the scope of fast‐forward GAB tools, including genome sequencing efforts, quantitative trait loci (QTL) mapping, genome‐wide association studies (GWAS) investigations, genomic selection (GS), haplotype‐based breeding (HBB), epigenomics, single‐cell genomics, pan‐genomics and transgenomics for harnessing the genetic landscape for improving SST in plants. The integration of HTP and AI and overcoming phenotyping bottlenecks are also discussed. A new breeding pipeline has been proposed that integrates single‐cell/tissue‐based genomics and phenotyping for fast‐tracking the designing of high‐yielding salinity‐smart cultivars. We anticipate that GAB can play a dynamic role in sustainable agriculture in the face of soil salinity by developing novel genetic resources to fast‐track the breeding of salinity‐smart crops and to maintain food security and feed growing populations.

## How does soil salinity hinder sustainable crop production? Appraisal on effects and tolerance strategies

Soil salinity poses a massive challenge to sustainable crop production by disrupting several morphological, physiological, biochemical, molecular and cellular mechanisms/responses, which are critical for plant growth and development. The tolerance or sensitivity of a specific crop depends on its ability to extract water and nutrients from saline soils, prevent the accumulation of toxic salt ions in metabolically active cellular compartments and prevent oxidative damage and disturbance to its metabolism (Melino and Tester, 2023; Morton *et al*., 2019; Munns *et al*., 2020; Negrão *et al*., 2017; Rawat *et al*., 2022; Raza *et al*., 2023d; Shabala and Munns, 2017; Shelden and Munns, 2023; Venkataraman *et al*., 2021; Zhao *et al*., 2020). Fast‐forward breeding and management approaches are needed to incorporate these traits and improve SST in crop plants.

All major food crops are highly sensitive to salinity, creating a threat to global food security. For instance, rice and wheat, which each offer ~40% of all calories consumed by humans, undergo mean yield penalties of ~70% and 40%, respectively, under high salinity conditions (Shabala and Munns, 2017). Refer to Table S1 for some recent examples of yield penalties. Sustainable agriculture relies on the competence of plant breeders to design high‐yielding salinity‐smart staple crop varieties, specifically contemplating climate change and intensified dependence on irrigation. However, current breeding approaches for SST largely focus on reducing sodium (Na^+^) uptake by plants, as Na^+^ toxicity severely influences plant performance under salinity conditions. This means picking low Na^+^‐accumulating plant varieties as breeding donors and phenotyping genotypes with lowered shoot Na^+^ content (Munns *et al*., 2012). Modern crop varieties can exclude between 90% and 95% of all Na^+^ extracted by roots (Shabala and Munns, 2017). Now, a critical question is: how can we move beyond the conventional focus on Na^+^ exclusion to develop truly salinity‐smart crop plants? This strategy, however, comes with a major caveat. The Na^+^ accumulation in the root zone establishes local salinity ‘hot spots’ near the root, inhibiting water uptake because of osmotic stress. Osmotic adjustment requires a substantial investment of carbon for osmotic regulation, leading to extensive yield losses (Munns *et al*., 2020; Roy *et al*., 2014). Furthermore, continuous Na^+^ deposition with irrigation water gradually enhances soil salinity, portraying it as unacceptable for the cultivation of major staple crops (Liu *et al*., 2020). While exclusion approaches may suggest short‐term solutions, they do not guarantee long‐term food security during ongoing climate change. Therefore, we need to understand the complex gene networks associated with main SST‐related traits, including shoot ion exclusion, shoot/root tissue tolerance and osmotic tolerance, to fast‐track future breeding programs (Munns and Tester, 2008; Roy *et al*., 2014). Now, a key question is: which key regulatory genes and pathways should be prioritized to fast‐track the breeding of salinity‐smart crops?

An alternative and potentially more sustainable approach involves improving plant tissue SST rather than simply excluding Na^+^ (Munns *et al*., 2016). During the mid‐1990s, the concept of osmoprotectant engineering was once considered a promising strategy to enhance stress tolerance (Hanson *et al*., 1994; Hanson and Burnet, 1994; Rontein *et al*., 2002). However, it was later found to be not viable due to the high carbon cost of *de novo* synthesis of organic osmolytes (Shabala and Shabala, 2011). Though plants must regulate osmotic potential in the cytosol using organic osmolytes, the bulk of osmotic adjustment should depend on inorganic ions, especially Na^+^ and Cl^−^, stored in vacuoles (Shabala and Shabala, 2011). In this context, an ‘ideal’ plant for salinity conditions would maximize NaCl sequestration in the vacuole while maintaining high cytosolic K^+^ levels and moderate organic osmolytes to ensure metabolic stability. Achieving this balance requires (1) efficient Na^+^ sequestration into the vacuole via *NHX*‐type Na^+^/H^+^ antiporters, (2) minimal Na^+^ leakage from the vacuole by controlling FV‐ (fast activating vacuolar) and SV (slowly activating vacuolar)‐type channels and (3) the retention of K^+^ in the cytosol by regulating voltage‐ and ROS‐gated K^+^‐permeable channels (Shabala and Shabala, 2011). Nevertheless, it should be highlighted that these physiological mechanisms are critical for short‐term salinity adaptation, and their long‐term sustainability remains doubtful, specifically under prolonged high‐salinity conditions. This raises a key question: can targeted genetic modifications of Na^+^ transporters adjust SST strategies under high‐salinity conditions?

Recent findings suggest that contrary to the assumptions above, the SOS1/NHX7 protein associated with Na^+^ extrusion at the plasma membrane may also contribute to vacuolar Na^+^ sequestration. High‐resolution imaging techniques and genomic‐based examinations have discovered that SOS1 localizes not only in the plasma membrane but also in late endosomes/prevacuoles and the vacuolar membrane (Ramakrishna *et al*., 2025). Similarly, a recent study on the halophytic species *Salicornia bigelovii* has shown that a homologue of SOS1 (SbiSOS1) is localized in the tonoplast, actively pumping Na^+^ into the vacuole to prevent cytosolic toxicity (Salazar *et al*., 2024). These findings challenged previous assumptions and opened new opportunities for breeding and engineering salinity‐smart crops by targeting intracellular Na^+^ distribution mechanisms. Nonetheless, given the complex and dynamic nature of salinity stress, a comprehensive systems‐level approach integrating HTP, AI and ML may offer a more accessible and predictive background for breeding crops with enhanced SST. By leveraging these cutting‐edge strategies, we can optimize selection strategies, identify novel trait combinations and fast‐track the designing of stress‐smart cultivars for saline‐affected environments. Now the question is: how can these novel insights into SOS1 localization and AI‐driven breeding strategies be translated into improved crop varieties?

Moreover, in natural‐field conditions, salinity stress rarely occurs in isolation. It frequently co‐occurs with other abiotic stresses, such as heat or nutrient deficiencies (called ‘combined abiotic stresses or multifactorial stress combination’), that further increase its negative impact on plant growth and productivity (Raza *et al*., 2025b; Rivero *et al*., 2022; Zandalinas *et al*., 2021a, 2024). For instance, excessive irrigation with brackish water (a common source of salinity stress) can lead to nutrient imbalances, shrinking the accessibility of essential minerals such as potassium and calcium, thus impairing plant stress responses (Shabala and Munns, 2017; Syvertsen and Garcia‐Sanchez, 2014). Similarly, heat stress can intensify the effects of high salinity by altering cellular homeostasis, increasing ion toxicity and accelerating water loss from plant tissues (Mesa *et al*., 2024; Rivero *et al*., 2014). The simultaneous occurrence of these stresses (i.e. salinity + heat, salinity + nutrient imbalances or salinity + heat + nutrient imbalances) presents a significant challenge for crop breeding, as plants must develop tolerance against combined abiotic stresses to withstand complex field conditions (Raza *et al*., 2025b; Zandalinas *et al*., 2024). Consequently, breeding and panomics strategies should not only target SST but also consider stress combinations to design strong climate‐smart crop plants (Raza *et al*., 2025b).

The above arguments highlight how high salinity impairs the growth and production of major crops, stressing the need for plant breeders to use fast‐forward breeding tools to design and breed climate‐smart future cultivars to safeguard yields and meet the market food demands.

## Modern genomic tools for designing future salinity‐smart crop plants: Are we on the right track for sustainable agriculture?

**Figure 2:**
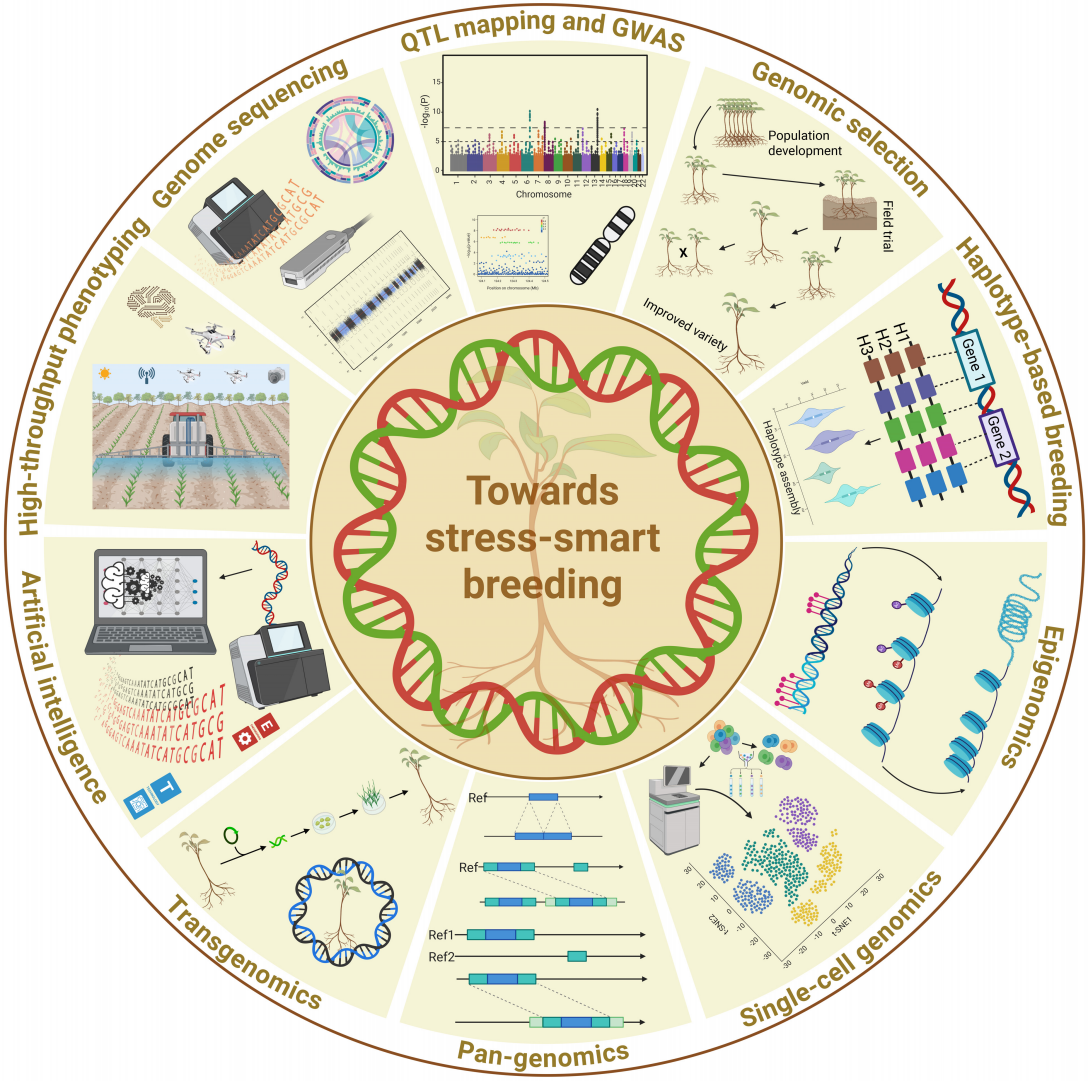
Comprehensive application of fast‐forward genomics tools for stress‐smart breeding within the framework of GAB for designing salinity‐smart plants. Utilizing these diverse genomics tools allows breeders to decode the genetic basis of salinity tolerance, discover essential genes and genomic regions accompanying stress tolerance and develop strategies for integrating these traits into crop fast‐forward breeding programs. This integrated, fast‐forward concept harnesses the power of genomics to fast‐track the design of stress‐smart crop varieties capable of thriving in stress conditions, eventually contributing to international food security and sustainable agriculture. GAB, genomics‐assisted breeding; GWAS, genome‐wide association studies; QTL, quantitative trait loci.

GAB integrates genomic resources, tools and molecular/DNA markers to fast‐track plant breeding, particularly for stress tolerance traits (Bohra *et al*., 2020; Varshney *et al*., 2005, 2021c). The utilization of marker‐assisted backcrossing (MABC) in rice has delivered positive outcomes in enhancing SST. For example, *Saltol*, a major QTL for seedling stage SST, was successfully introgressed from the donor ‘FL478’ to ‘Pusa Basmati 1509’, resulting in salinity‐tolerant lines (Yadav *et al*., 2020). BC_3_F_4_ rice lines, developed through introgression of SST genes, mainly the *Saltol* QTL, showed improved SST (Maré *et al*., 2020). Similarly, salt‐tolerant lines derived from the adapted rice variety ‘Rassi’ displayed reduced yield loss in BC_3_F_2_ phase, with eight introgression lines showing promising results (Bimpong *et al*., 2016). Similarly, MABC enabled the transfer of *Saltol* to elite rice varieties, with background selection confirming the recovery of favourable traits for SST (Marè *et al*., 2023). MABC, together with physiological assessments, highlights the success of MABC in enhancing SST in rice (Marè *et al*., 2023). Likewise, MABC has been instrumental in pyramiding QTLs for tolerance in ‘Improved White Ponni’ rice variety against diverse stresses, including salinity (Muthu *et al*., 2020). Earlier, James *et al*. (2012) established the successful integration of *TmHKT1;5‐A* (*Nax2* locus) from *Triticum monococcum* (einkorn wheat) into durum wheat via MABC, and subsequent progeny (BC_4_F_4_ lines) showed improved yield potential under salinity conditions. On the most saline site, lines containing *TmHKT1;5‐A* (*Nax2*) yielded 25% more than the cultivar without the *Nax2* locus (James *et al*., 2012; Munns *et al*., 2012). Field trials in Bangladesh showed that, when crossed into adapted Bangladeshi bread wheat lines, the *Nax* genes could increase grain yield by 10%–20% on high‐salinity soils (James *et al*., 2023). For more previous success stories for designing salinity‐smart wheat (Kotula *et al*., 2024) and other crops, see the authoritative review of Melino and Tester (2023). Nevertheless, these examples highlight the success of conventional breeding combined with molecular markers for specific traits. However, these advances often demand extensive time and resources, mainly when dealing with multigenic traits such as SST. Modern genomic‐driven breeding tools deliver a more effective and specific alternative by allowing the synchronized selection and manipulation of multiple traits. These tools integrate high‐throughput genotypic and phenotypic data to fast‐track breeding cycles, reduce the time required for variety development and boost the precision of trait selection. Figure 2 highlights how these modern genomic tools can complement conventional approaches for designing future salinity‐smart crop cultivars for sustainable agriculture.

Beyond the hypothetical potential of GAB tools, their practical use in plant breeding programs has fruitfully augmented the development of new salinity‐smart cultivars. As discussed in the above paragraph (MABC) and in subsequent GS and HTP sections, these tools have been successfully employed to improve SST and also helped identify salinity‐smart genotypes at large scales. These cases prove how breeders integrate genomic tools into practical breeding pipelines, ensuring that newly developed cultivars meet both yield and tolerance targets.

Despite their promise, the widespread application of these advanced genomic tools in breeding programs faces challenges, particularly in resource‐limited regions (Varshney *et al*., 2021c). The high costs associated with genome sequencing, genotyping platforms and bioinformatics setup can limit access for breeding programs in developing countries. Furthermore, the need for well‐trained personnel and high‐performance computing resources further limits the practical adoption of these tools in such regions. A key question is whether the use of GAB tools remains restricted to large multinational companies or whether fundamental genomics approaches, such as marker‐assisted selection (MAS) and low‐cost genotyping‐by‐sequencing, can be adopted more largely by national breeding programs and small‐scale breeding companies. Addressing these constraints will require increased international collaborations, technology transfer initiatives, and the development of cost‐effective genomic approaches designed to meet the requirements of resource‐limited areas. For example, recent advances in portable sequencing technologies, such as Oxford Nanopore MinION (Dumschott *et al*., 2020), and the growing accessibility of open‐source bioinformatics tools/databases (*some are highlighted in the conclusion section*) have made it viable for smaller breeding programs to apply basic genomic selection strategies with minimal infrastructure.

To further illustrate the practical considerations for employing GAB tools, Table S2 summarizes the infrastructure, equipment and expertise required for different levels of GAB applications. It categorizes these tools into three levels, that is, basic, intermediate and advanced, according to their accessibility across different breeding programs.

### Advances in genome sequencing of naturally salinity‐tolerant plants: Time to act and deliver salinity‐smart crop plants

Since the first *Arabidopsis thaliana* reference genome in 2000, advancements in sequencing and computational technologies have guided the sequencing of >1144 genomes from 782 plant species by 2020 (Xie *et al*., 2024). During 2021–2023, 2373 genomes have been assembled from 1031 plant species, demonstrating a great leap (Xie *et al*., 2024). These genomes offer valuable resources for studying plant genomics, genetics, evolution and breeding for trait and stress tolerance improvement.

**Figure 3:**
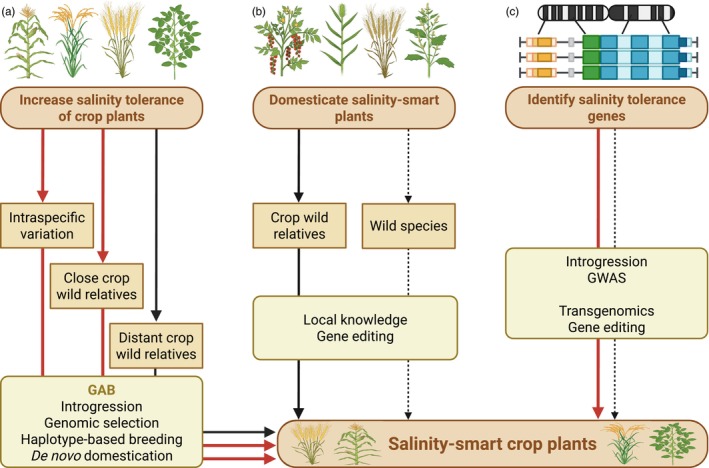
Strategies to act and deliver salinity‐smart crop plants. (a) Enhancing salinity tolerance in cultivated crop plants involves leveraging intraspecific variation and incorporating genetic diversity from both close and distant CWRs through GAB methods. (b) Gene editing technologies and local knowledge enable the domestication of naturally occurring salinity‐smart plants, including CWRs and wild species. (c) Identifying salinity tolerance‐associated genes guides transgenomics and gene editing efforts in designing salinity‐smart crop plants. In this context, halophytes and CWRs serve as valuable sources for isolating key genes for integration into modern cultivated crops or introgression into sensitive cultivars. In this scheme, GWAS can also be employed if the ‘missing gene’ is still there but has become non‐functional because of a mutation. For more information, we recommend reading the main text and the authoritative review of Melino and Tester (2023). Red arrows represent strategies that have already produced results, solid black arrows represent promising strategies that can guide the near‐future delivery, and dotted arrows represent strategies with potential for distant future delivery. Modified from Melino and Tester (2023) under CC BY 4.0 international licence. CWRs, crop wild relatives; GAB, genomics‐assisted breeding.

The freely available genomic sequences of many major food crops, such as rice, maize, wheat, sorghum, chickpea, soybean, peanut, peas and many more (https://phytozome‐next.jgi.doe.gov/; https://www.ncbi.nlm.nih.gov/genome/browse#!/overview/), provide a base for modifying their genetic makeup to control stress‐smart breeding mechanisms and adjust their functions (Figure 3). In addition to these major food crops, naturally salinity‐tolerant plants, such as wild relatives of crops, halophytes and mangroves, offer exceptional opportunities for enhancing SST in major crops (Table 1). Unlike traditional breeding approaches that primarily focus on salt exclusion, leveraging the genetic diversity of these naturally salt‐loving plants could enable the integration of key halophytic traits into modern crops, promoting sustainable agriculture in saline environments (Liu *et al*., 2020; Melino and Tester, 2023).

**Table 1:** Sequenced genomes of some naturally salinity‐tolerant plants and their significance to salinity stress tolerance

Plant category	Specie name	Genome size (Mb)	Number of genes	Key features and SST insights	References
Wild relatives	*Oryza coarctata* (rice)	~570	33 627 and 4916 TFs	Halophytic rice species with high salinity and submersion tolerance Salt exclusion	Mondal *et al*. (2018); Zhao *et al*. (2023)
	*Triticum dicoccoides* (emmer wheat)	10.5 Gb	62 569	Source of salinity‐tolerant alleles for modern wheat improvement	Avni *et al*. (2017)
	*Solanum habrochaites* (tomatoe)	950.7	33 567	Wild tomato species with multiple stress‐responsive genes for the advancement of modern cultivated tomatoes	Yu *et al*. (2022)
	*S. galapagense* (tomatoe)	859.9	33 108		Yu *et al*. (2022)
	*S. pennellii* (tomatoe)	1.2 Gb	44 966		Bolger *et al*. (2014)
	*S. chilense* (tomatoe)	∼914	32 972		Molitor *et al*. (2024); Stam *et al*. (2019)
	*Paspalum vaginatum* (paspalum)	593	45 843	Bioenergy crop with strong SST	Sun *et al*. (2022)
	*Ipomoea pes‐caprae* (sweet potato)	1.05 Gb	34 077	Source of salinity‐tolerant genes and transposable element‐induced diversification Key SST mechanisms include ion transport, DNA stability and medicinal compound synthesis under salinity stress	Cheng *et al*. (2023b)
Halophytes	*Thellungiella parvula*	~140	30 419	Model extremophile for understanding SST mechanisms	Dassanayake *et al*. (2011)
	*Thellungiella salsuginea*	~233.7	28 457	Defend themselves against stress via ionic and osmotic equilibrium, ABA signalling and wax production	Wu *et al*. (2012)
	*Eutrema salsugineum*	241	26 531	Model extremophile plant Ion transport adaptations	Yang *et al*. (2013)
	*Cakile maritima*	719	na	Maintain water status and osmotic balance Selective K^+^ uptake Functional integrity of photosynthetic systems	Debez *et al*. (2013)
	*Suaeda fruticose*	na	54 526	A highly salinity‐tolerant succulent shrub, accumulating Na^+^ and Cl^−^ without salt glands or bladders SST via ion homeostasis and osmoprotectants	Diray‐Arce *et al*. (2015)
	*Halogeton glomeratus*	~0.95 Gb	50 267	Highly salt‐tolerant halophyte thriving in arid and saline deserts Adapts through succulent stems and leaves, salt compartmentalization and specialized stomatal regulation	Wang *et al*. (2015); Xiangling *et al*. (2023)
	*Suaeda aralocaspica*	452	29 604	C_4_ halophyte adapted to extreme saline deserts	Wang *et al*. (2019b)
	*Suaeda salsa*	447.98	27 927	A leaf/stem succulent halophyte with extreme SST, driven by genetic evolution Nutrient‐rich vegetable	Cui *et al*. (2024a)
	*Suaeda glauca*	1.02 Gb	54 761	Annual halophyte with extreme SST Succulent leaves for salt sequestration It lacks salt glands or bladders, relying on parenchymatous tissue and giant vacuoles for salt and water storage	Cheng *et al*. (2023a)
	*Mesembryanthemum crystallinum*	~369	24 204 transcripts	Thrives in extreme salinity, switching from C_3_ to CAM photosynthesis under salinity and drought Model for studying SST and photosynthetic adaptation mechanisms	Sato *et al*. (2024)
	*Puccinellia tenuiflora*	1.107 Gb	39 725	Thrives in extreme salinity and alkaline soils Model for improving salinity and drought tolerance in cereal crops	Guo *et al*. (2020)
	*Aeluropus littoralis*	354	15 916	Stress‐adapted halophyte with osmoprotectant mechanisms and extreme SST Secretes salt through specialized salt glands and serves as a valuable genetic resource for crop SST	Hashemi‐Petroudi *et al*. (2022)
	*Limonium bicolor*	2.92 Gb	48 690 transcripts	Model recretohalophyte with salt‐secreting glands for extreme SST Features autofluorescent salt glands	Yuan *et al*. (2022)
	*Aeluropus lagopoides*	~ 400	592 893 transcripts	Thrive in extreme salinity environments A salt‐tolerant C_4_ perennial grass with a high salt secretion ability SST mechanisms involve ion excretion and osmotic homeostasis	Bosamia *et al*. (2024)
Mangroves	*Kandelia obovata*	177.99	19 138	Thrives in extreme salinity tidal wetlands Protect biodiversity and combat erosion Evolutionary traits, including flowering and SST	Hu *et al*. (2020)
	*Avicennia marina*	~457	31 477	Thrives in seawater Possesses multiple salinity‐responsive genes	Natarajan *et al*. (2021)
	*Bruguiera gymnorhiza*	309	34 403	Epigenetic regulation for salinity tolerance and adaptation	Miryeganeh *et al*. (2022)
	*Avicennia rumphiana*	499.6	37 347	Secretes salt through its leaves Salt excretion mechanisms	Shearman *et al*. (2025)

na, not available; SST, salinity stress tolerance (SST).

Crop wild relatives (CWRs) represent invaluable reservoirs of SST genes, which have been largely lost in modern cultivars due to domestication. *De novo* domestication methods offer new ways forward for designing superior crop varieties by leveraging the genetic diversity of CWRs (Bohra *et al*., 2022a, 2022b; Khan *et al*., 2020). The idea of integrating CWRs into breeding programs to improve SST in modern crops has been debated for decades (Colmer *et al*., 2006; Flowers and Yeo, 1995; Kotula *et al*., 2024; Palmgren and Shabala, 2024; Venkataraman *et al*., 2021). Notably, species such as *Oryza coarctata* (rice), *Triticum dicoccoides* (wild emmer wheat), wild tomato species (*Solanum habrochaites*, *S. galapagense*, *S. pennellii* and *S. chilense*) and *Paspalum vaginatum* (a salt‐tolerant grass) harbour important genetic traits for SST (Table 1). Despite their potential, integrating these wild genomes into breeding programs remains challenging due to genetic incompatibilities and undesirable agronomic traits. However, advances in next‐generation sequencing (NGS), GWAS and CRISPR‐based genome editing provide novel opportunities to determine and introduce beneficial SST genes into cultivated crops. For example, wild relatives of rice (Mondal *et al*., 2018; Zhao *et al*., 2023) and wheat (Avni *et al*., 2017; Feng *et al*., 2018; Shavrukov *et al*., 2010) possess key regulatory elements that enhance ion homeostasis and osmotic balance under high salinity. Identifying and utilizing such genes could significantly improve SST in staple crops.

Halophytes are naturally adapted to survive in saline environments, making them valuable models for understanding SST mechanisms (Flowers and Colmer, 2015; Kotula *et al*., 2024; Melino and Tester, 2023; Munns *et al*., 2020; Rawat *et al*., 2022; Venkataraman *et al*., 2021). Over the past decade, genomic studies on halophytes have provided insights into their unique adaptations, including specialized ion transport mechanisms, osmoprotectant accumulation and stress‐responsive gene networks (see Table 1 for more details on their genome insights). Some key species, such as *Eutrema salsugineum*, *Suaeda aralocaspica*, *Puccinellia tenuiflora* and *Aeluropus littoralis*, exhibit distinct molecular strategies to withstand extreme salinity (Table 1). Notably, some halophytes share close genetic relationships with major crops, facilitating gene transfer via GAB or transgenomic approaches. For instance, *Puccinellia tenuiflora*, a salt‐tolerant grass closely related to cereals (Guo *et al*., 2020), offers unique genetic resources for enhancing SST in wheat and barley. By leveraging the unique genetic makeup of these naturally tolerant species, we can enhance the SST of major food and grain crops through genetic manipulation and transgenomics breeding‐based strategies. By doing this, traits from halophytic ancestors have enhanced SST in conventional crops, but a single‐gene approach is not enough due to the complexity of SST, as reviewed by Rawat *et al*. (2022). Overall, these resources present an exciting opportunity to improve crop SST and ensure food security in regions affected by high soil salinity (Figure 3).

Mangroves, which have evolved over millions of years under extreme conditions, including high salinity and temperatures and hypoxia, offer a unique perspective on plant stress adaptation. As reviewed by Xie *et al*. (2024), so far, 40 high‐quality mangrove genomes are available, shedding light on stress tolerance mechanisms. As case studies, high‐quality genomes of species, such as *Bruguiera gymnorhiza* (Miryeganeh *et al*., 2022) and *Avicennia marina* (Natarajan *et al*., 2021), have discovered key epigenetic and genetic mechanisms underlying SST (Table 1). These findings not only improve our understanding of SST but also provide potential targets for engineering crops with enhanced tolerance to both salinity and waterlogging (either single or combined), as these stresses often coincide in coastal and flood‐prone agricultural regions.

The priority to enhance crop SST is compelling, yet opportunities thrive. Nevertheless, there remains a gap in delivering salinity‐smart crops to breeders and detailing such achievements. Genetic improvement using intraspecific variation or close CWRs is viable under low salinity levels but demands more radical methods. Introducing modern genomic and genetic methods offers ways to introgress SST from distant CWRs or domesticate salinity‐smart wild species. This requires shifting from traditional crops to advancing novel varieties from plants with natural power to thrive under high salinity conditions (see Figure 3 for more details).

We propose that the genetic potential of these naturally high salinity‐tolerant sources should be fully exploited using panomics tools (Raza *et al*., 2025b). By introgression via GAB methods or transferring their essential genes/regions into cultivated crops using transgenomics or gene editing tools, we can design salinity‐smart future crops capable of thriving under challenging conditions (Figure 3).

### Key insights from QTL mapping and GWAS‐based investigations: Leveraging genetic mechanisms underlying salinity tolerance

GAB integrates genomic mapping to develop improved cultivars by identifying QTLs and genomic regions associated with desired traits such as SST in plants (Bohra *et al*., 2020; Kumar *et al*., 2022; Varshney *et al*., 2005, 2021c). Generally, SST is a complex trait controlled by multiple genes and has been widely examined using diverse molecular markers (Table 2). Likewise, QTL mapping and association mapping approaches have substantially expanded our comprehension of SST‐associated traits (Table 2). Below, we have discussed some recent wheat and rice studies identifying key QTLs/genes associated with SST. Table 2 provides a comprehensive list of the most recent examples of some major crops, such as rice, wheat and barley.

**Table 2:** Some genomic studies that identify genomic regions, molecular markers, genes and QTLs associated with salinity tolerance in crops

Stress condition	Populations	Number of mapping individuals	Genotyping approach	Map distance (cM)	Number of mapped markers	Number of QTLs/genes	Chr location	Key findings	References
Rice (*Oryza sativa* L.)									
NaCl, CaCl_2_ and MgSo_4_ in water with ECe–40 mmol; till maturity	F_2_ RILs (PS5 × CSR10)	140	SSR markers	1641	436 rice HvSSR and 30 SSRs	39 QTLs	All 12 Chr	39 QTLs identified under salinity stress at the reproductive stage *qNaL‐1.2*, *qNa/KL‐1.3*, *qKR‐1* and *qNa/KL‐1.2* were found in roots and leaves Novel QTL *qGY*‐*2* was also identified for grain yield Four QTLs were found for Chl content	Pundir *et al*. (2021)
100 mM NaCl; 14 d	Diverse rice varieties	155	SNP markers	na	33 864 SNPs	151 MTAs	1, 2, 4, 6, 7, 8, 9, 10, 11 and 12	151 MTAs identified on 10 chromosomes *Os01g0304100* was identified as encoding a cation chloride cotransporter *Os01g0624700* and *Os01g0812000* encode a WRKY transcription factor *Os02g0730300* identified as a high‐affinity K^+^ transporter	Nayyeripasand *et al*. (2021)
80 mM NaCl; 10 d	F_1_ RILs (Huazhan × Nekken2)	120	SNP markers	na	4858 SNPs	16 QTLs	3, 4, 6, 8, 9, 10, 11 and 12	At the bud stage, 16 SST‐related QTLs were detected A major effect candidate QTL (*qST12.3*) and gene (*LOC_Os12g25200*) found to be associated with SST	Yin *et al*. (2023)
10 ds m^−1^ underground brine; 7 d	F_2_ RILs (Jileng 1 × Milyang 23)	253	SNP markers	1408.03	2921 recombination bin markers	12 QTLs	2, 3, 4, 6, 8 and 11	Five QTLs were found to be environmentally stable under salinity stress, and their roles were confirmed by transcriptome analysis One key gene *OsRCI2‐8* (*Os06g0184800*) is responsible for SST	Geng *et al*. (2023)
125 mmol L^−1^ NaCl; 6 d	Diverse accessions	182	WGR	na	402 InDels	28 allelic loci	All 12 Chr	A total of 14 SST‐related InDels were discovered 28 allelic loci explaining 6.83–11.22% of the PV Haplotype analysis spotted six InDels accompanying SST‐related traits	Yang *et al*. (2023a)
50 mmol L^−1^ NaCl for 3 days and 120 mmol L^−1^; 7 d	RILs (KY131 × XBJZ)	195	10 K Array GBTS	1874.85	527 bin markers	14 QTLs	1, 2, 3, 4, 7, 9, 10, 11 and 12	Three QTLs (*qSST12‐1*, *qSST12‐2* and *qRSL12*) were co‐positioned in the interval C12_17379052–C12_17519826 on Chr 12, covering 16 candidate genes for SST	Li *et al*. (2023)
150 mM NaCl; 4 d	F_2_ (SR86 × Nipponbare) and (SR86 × 9311)	30 salt‐tolerant and 30 salt‐sensitive	QTL‐seq and BSA‐seq	na	na	11 genes	na	Eleven SST‐related candidate genes were located Two candidate genes *LOC_Os04g03320.1* and *BGIOSGA019540* were highly expressed and are critical for SST	Gao *et al*. (2023)
150 mM NaCl; 7 d	Natural population	130 indica and 81 japonica accessions	50 K rice gene SNP microarray	na	36 727 SNPs	9 major QTLs	1, 3, 4 and 5	Identified QTLs are located in the same region as the *OsMDH1*, *OsSRFP1* and *OsCDPK7* genes *qRSL1‐2* was discovered to harbour 18 genes associated with SST	Li *et al*. (2024a)
0.3% and 0.5% NaCl (w/v); 3, 4, 7 and 10 d	Diverse accessions	541	SNP markers	na	302 900 SNPs	3 QTLs	3, 4 and 5	*qGRG3‐1*, *qGRG3‐2* and *qGRG4* were identified as key QTLs for SST A candidate gene *OsTMF* in *qGRG3‐2* is associated with SST	Liu *et al*. (2024)
Wheat (*Triticum aestivum* L.)									
0.2, 0.4, 0.6, 0.8 and 1 salt solution (standard concentration of seawater; 7 d)	31 local landraces, 126 exotic and 150 cross‐derived cultivars	307	KASP markers and Wheat 660 K SNP array	4424.4	402 176 SNPs	3 QTLs	1A, 3B, 6B	*QSt*.*nwafu*‐*6B* identified as a novel QTL 53 genes were found to be regulating SST	Yu *et al*. (2020)
0.18 and 0.3% mM NaCl; till maturity	Five varieties, 10 landraces and 176 improved cultivars	191	Axiom wheat 660 K SNP array	na	322 590 SNPs	11 QTLs	1B, 3B, 4A, 4D, 5A, 5B and 7A	11 loci were identified for different traits under salinity stress Three out of 14 KASP marker loci were confirmed against yield‐related traits associated with SST	Hu *et al*. (2021)
0.18% and 0.3% mM NaCl, 240 days (whole‐growing season)	F_7_ RILs (Zhongmai 175 × Xiaoyan 60)	350	Wheat 55 K SNP array	3250.71	53 063 SNPs	90 stable QTLs	All Chr except 4D, 6B and 7D	90 stable QTLs were identified, out of which eight were validated in a natural population *QPh‐4B* was confirmed as an allele of *Rht‐B1*	Luo *et al*. (2021)
1.2% (w/v) NaCl; 7 and 10 d	Diversity panel and DH lines	323 accessions and 150 DH lines	Wheat 660 K SNP array	4082.4	395 675 SNPs	91 QTLs and 269 loci	3D, 5B, 3B, 4D	A total of 269 loci for SST were identified Fourteen overlapped QTLs were identified with a PV of 10% *TaRN1* and *TaRN2*, two novel candidate genes were identified against salt stress	Li *et al*. (2021b)
100 mM NaCl; 13 d	F_2_:F_6_ RILs (Excalibur × Kukri)	128	GBS	3084	3236 SNPs	9 QTLs	1A, 2A, 2B 3A, 5A, 7B and 2DS2	Novel QTLs for shoot ion‐independent tolerance (*QG*(*1–5*).*asl*‐*7B*), Cl^−^ accumulation (*QCl.asl*‐*3A*) and K^+^: Na^+^ DW (*QK*:*Na.asl*‐*2DS2*)	Asif *et al*. (2021)
High salinity soils	Elite spring lines	289	90 K SNP array	na	15 737 SNPs	118 and 120 MTAs under low and high salinity	1A, 1B, 5A, 2B, 7A, 6B, 6A, 7D, 2A, 4B, 3D, 5B and 7B	High degree of major linkage disequilibrium (>52%) was noticed between SNPs on diverse chromosomes, representing epistatic interaction Salt stress index displayed a positive significant relationship to grain yield/plant	Alotaibi *et al*. (2022)
100 mM NaCl; 3 weeks	Diverse cultivars and landraces	298	GBS	na	46 203 SNPs	29 MTAs	All 21 Chr	A total of 29 functional MTAs were spotted 27 candidate genes were associated with SST	Javid *et al*. (2022)
50, 100 and 150 mM; 40 d	Diverse varieties and landraces	125	DArT‐seq	na	4417 SNPs	5 loci	6B, 6D, 7A and 7D	Effective 4417 SNPs encircling all the Chr with marker 37%, 32% and 31% density in B, D and A genomes, respectively Five loci were found to be associated with diverse traits under salinity	Khan *et al*. (2022)
0.08, 0.26, 0.37 and 0.38% salt	F_2_‐F_6_ RILs (EP × HMM)	827	90 K SNP array	4128.9	9053 SNPs	1 novel QTL	All 21 Chr	A novel QTL (*QSt.nftec‐2BL*) was discovered on Chr 2B for SST Plants harbouring *QSt.nftec‐2BL* produced increased grain yields by upto 21.4%	Zhang *et al*. (2023d)
125 mM NaCl; 35–36 days after sowing	Diverse accessions	580	SNP markers	na	883 300 SNPs	95 QTLs	Across 19 Chr	Of 95 QTLs, 54 were novel and 41 intersected with earlier discovered QTLs Identified QTLs were associated with diverse SST‐related traits 58 502 haplotype blocks were found	Pasam *et al*. (2023)
Barley (*Hordeum vulgare* L.)									
150 mM NaCl; 2–4 d	Diverse accessions	350	DArTseq and SSR markers	na	∼24 000 SNPs	19 QTLs	H1, H2, H3, H4, H5, H6, H7	19 QTLs with 52 SST‐associated markers were identified L6H495910722, L6H286731484 and L7H614807240 marker alleles have a positive phenotypic effect	Mwando *et al*. (2020)
200 mM NaCl; 12 d	Diverse accessions	121	9 K SNPs array	na	7842 SNPs	11 QTLs	1H, 2H, 3H, 4H, 5H, 6H, and 7H	Key genomic regions harboured 1500 candidate genes New candidate genes (*ATK2* on Chr 1H, and *Squamosa promoter‐binding‐like protein 6* at Chr 5H) were discovered associated with SST	Thabet *et al*. (2021)
75, 90, 120 and 150 mM NaCl; 14 d	103 DH lines (CM72 × Gairdner) and 85 diverse accessions	103 DH lines and 85 barley germplasm	DArT and SSR markers	na	350 DArT and 84 SSRs	6 QTLs	1H, 3H and 4H	Identified six stable QTLs Shoot length (65.6% and 50.3%) was affected most among all phenotypic traits QTL on 3H detected more linked with SST	Mwando *et al*. (2021)
150 mM NaCl; 10 d	Diverse accessions	208	SNP markers	na	10 323 SNPs	38 putative QTLs and 153 high‐confidence predictive genes	1H, 2H, 3H, 4H, 5H, 6H and 7H	38 highly significant SNPs were identified associated with SST Two SNPs on Chr 1H, two on Chr 3H and one on Chr 4H were notably associated with seedling fresh and dry weight under salt stress SST‐related QTL associated with marker 3_599466174 was situated on Chr 3H	Sayed *et al*. (2022)
300 mM NaCl; 2 weeks	Diverse accessions	214	DArT and SNP markers	na	17 447 DArT/SNPs	2 major and several minor QTLs	1H, 2H, 3H, 4H, 5H, 6H and 7H	One major QTL (*Q.SL.2H*) associated with SST was detected on Chr 2H Another major QTL (*Q.K.4H*) was detected on Chr 4H that overlapped with *HvHKT1;5* gene and was responsible for K^+^ retention in leaves Several minor QTLs were also discovered, assisting the selection of salinity‐tolerant cultivars	Zhu *et al*. (2022a)
300 mM NaCl; 3 weeks	Core collection	288	50 K SNP array	na	25 342 SNPs	5 candidate genes	2, 4, 5, 6 and 7	Among SNP pool, 54 significant SNPs were identified and distributed on 7 Chr Chr 4, 6 and 7 are a ‘hot‐spot’ region harbouring SST‐related genes *PGK2*, *BASS3*, *SINAT2*, *AQP* and *SYT3* are key genes for SST	Xu *et al*. (2023b)

BSA‐seq, bulked segregant analysis‐sequencing; Chr, chromosome; DArT, diversity array technology; DH, double haploid; GBS, genotyping by sequencing; GBTS, genotyping by target sequencing; KASP, kompetitive Allele‐Specific PCR; MTA, marker‐trait associations; PV, phenotypic variation; QTL‐seq, quantitative trait locus‐sequencing; RILs, recombinant inbred lines; SSRs, simple sequence repeats; SNPs, single nucleotide polymorphisms; WGR, whole‐genome resequencing. The ‘na’ means no data available in the article.

In wheat, QTL mapping discovered a significant QTL (*QSt.nftec‐2BL*) associated with SST, located on a 0.7 cM interval using new SSR markers (Zhang *et al*., 2023d). Selection for this QTL resulted in alleles linked to SST, which led to an increase in grain yields (up to 21.4%) compared with non‐selected counterparts, signifying its effectiveness in enhancing wheat yield under salinity conditions (Zhang *et al*., 2023d). Another wheat study by Turki *et al*. (2023) identified two major QTLs (*qDL4* and *qDL5*) related to SST. These QTLs notably affect the proportion of dead leaves, explaining 68% of the total phenotypic variation (PV), making them promising candidates for stress‐smart MAB programs (Turki *et al*., 2023).

In rice, 16 SST‐related QTLs were discovered at the bud stage, located on different chromosomes (Chr) (Yin *et al*., 2023). Among these QTLs, *qST12.3* was narrowed down to a 192‐kb region on Chr12 with the help of map‐based cloning. Moreover, a range of candidate genes associated with SST was discovered via QTL mining and assessment, and the findings indicated that *LOC_Os12g25200* could negatively regulate SST in rice (Yin *et al*., 2023), providing the source for further testing and designing of salinity‐smart rice varieties. In another study, Geng *et al*. (2023) identified 12 QTLs associated with different growth‐related traits and contributing to SST in rice. Of these, five QTL intervals were discovered as stable QTLs. Moreover, Pruthi *et al*. (2023) identified 17 and 28 QTLs for seedling and flowering stage traits, respectively. Additionally, they also identified candidate genes underlying SST at both stages, such as *OsHAK13*, *OsCYP21‐4*, *GIF1* and *OsRGG20* at flowering stage SST, whereas *OsHAK20*, *STRK1* and *OsMADS25* were identified at seedling stage SST. The gene expression analysis directed the upregulation of specific genes under salinity conditions at each stage. Stacking genes/QTLs for SST at both stages is important due to changes in genetic control, with emphasis on targeting required QTLs from donors while recalling valuable QTLs from the recurrent parent for MAS to boost SST in rice (Pruthi *et al*., 2023). Another study identified 14 QTLs from two different RIL populations located on Chr1/2/3/4/7/9/10/11/12. Of these, *qSST12‐1*, *qSST12‐2* and *qRSL12* were co‐specified in a 140‐kb overlap interval on Chr12, including 16 candidate genes accompanying SST in rice (Li *et al*., 2023).

GWAS offers a strong platform to discover contributory alleles for specific traits, aiding in improving SST in crop plants through GAB. By exploiting historical recombination events, GWAS has been influential in pinpointing genomic areas accountable for SST in different plant species (Table 2). For instance, Phan *et al*. (2023) identified four QTLs correlated with standard evaluation scores through GWAS analysis. Some QTLs co‐located with formerly discovered traits. Three candidate genes, including *LOC_Os01g23640*, *LOC_Os01g23680* and *LOC_Os01g71240*, were shortlisted for *qSES1.1* and *qSES1.3* regions affecting SST in rice. These discoveries offered worthy insights for future genetic improvement of rice under stress conditions (Phan *et al*., 2023). A recent study elaborated 402 insertion–deletion (InDel) polymorphic markers for japonica rice, assisting genetic diversity analysis and GWAS for SST‐associated traits (Yang *et al*., 2023a). Genetic diversity investigation discovered 1204 allelic variants, whereas GWAS recognized 14 SST‐related InDels on Chr1‐5, Chr9, Chr10 and Chr12, offering valuable tools for molecular discovery of salinity‐smart rice genotypes (Yang *et al*., 2023a).

A comprehensive GWAS investigation has discovered 12 unique QTLs (*qSES1*, *qSL1*, *qRL1*, *qSUR1*, *qSL8*, *qK8*, *qK1*, *Saltol*, *SKC1*, *OsSalT* and *salT*) associated with nine SST‐related traits in rice (Maniruzzaman *et al*., 2023). These novel QTLs are located on seven diverse Chr and are distinct from earlier detected SST‐related loci such as *Saltol*, *SKC1*, *OsSalT* and *salT*. Furthermore, they discovered 28 significant digenic/epistatic interactions between chromosomal regions, emphasizing complex genetic mechanisms underlying SST in rice. The authors suggest that integrating these novel, reliable and stable QTLs presents promising opportunities for designing salinity‐smart rice cultivars (Maniruzzaman *et al*., 2023). By combining GWAS and linkage mapping, Xu *et al*. (2023a) discovered three QTLs linked to shoot Na^+^ concentration (SNC) on Chr1, Chr4 and Chr12, whereas eight QTLs associated with shoot Na^+^/K^+^ ratio (SNK) were found on Chr1, Chr4, Chr6, Chr9, Chr10 and Chr12. Additionally, three QTLs for seedling survival rate (SSR) were located on Chr4, Chr8 and Chr10. Linkage mapping discovered five QTLs linked with SNC, SKC, SNK and SSR on Chr1, Chr4 and Chr12, with *qSKC12* and *qSNK12* considered the same QTL (*qSK12*) on Chr12. Moreover, the lead SNP (Chr12_20864157) correlated with SNK was found within this QTL. They also detected a 195‐kb region on Chr12, including the candidate gene *LOC_Os12g34450*, associated with SST in rice (Xu *et al*., 2023a). This study presents new perceptions of the genetic systems underlying SST in rice and guides future breeding programs for designing stress‐smart rice varieties.

The above‐discussed studies offer additional insights into the genetic basis of SST and present promising opportunities for designing stress‐smart cultivars via GAB. Future research efforts should focus on leveraging fast‐forward genomic tools for gene discovery, performing multi‐stage and multi‐environment assessments and nurturing collaborative corporations to translate genomic discoveries into practical solutions for stress‐smart sustainable agriculture and future food security.

### Prioritizing gene networks for salinity tolerance: A ‘horses for courses’ approach

Recent advances in plant genomics have highlighted the need to accept a ‘multi‐gene’ approach to design salinity‐smart crops (Rawat *et al*., 2022; Venkataraman *et al*., 2021). The complex nature of SST imposes the synchronized regulation of many genes, constituting a comprehensive ‘full gene package’ that directs diverse physiological mechanisms under salinity conditions. Now the question arises: how many genes are genuinely necessary in the ‘full gene package’ for breeders to manage efficiently? Can we handle three, five or even 10 genes concurrently? Will this number be sufficient to achieve the goal of salinity‐smart crops without yield penalties? As discussed earlier, rather than relying exclusively on single gene approach like *SalTol* or *HKT1;5*, reaching true tolerance includes a composed regulation of a network of genes (‘full gene package’) involved in osmotic adjustment, ion homeostasis, shoot/root tissue tolerance and stress signalling (Rawat *et al*., 2022; Raza *et al*., 2025b; Roy *et al*., 2014; Venkataraman *et al*., 2021).

The idea of a ‘silver bullet’ gene for SST has proven to be very simplistic. For instance, early candidates like the vacuolar Na^+^/H^+^ exchanger *NHX1* firstly displayed promising results but eventually failed to deliver constant results due to the lack of co‐expressed transport systems and the need for tight control of vacuolar channels and H^+^ pumps (Munns *et al*., 2020; Munns and Gilliham, 2015; Nguyen *et al*., 2019; Palmgren and Shabala, 2024; Shabala, 2017). Similarly, the SOS signalling pathway, including genes like *SOS1*, *SOS2* and *SOS3*, demonstrates the need for multifaceted gene regulation rather than single‐gene overexpression. Although transgenic plants overexpressing these genes exhibited limited recovery, their effectiveness remains context‐dependent, advising that the ‘full package’ of genes required for true SST remains mysterious (Yang *et al*., 2009).

It is also clear that SST is regulated at the cellular and tissue levels, and the expression of significant genes needs to be context‐specific. For instance, while *HKT1;5* transporters are crucial for Na^+^ exclusion from the shoot, their efficiency depends not only on salinity levels and plant species but also on their interaction with other ion transporters and signalling components, as reviewed by Venkataraman *et al*. (2021). Recent reports have explained that different *HKT1;5* haplotypes can confer either Na^+^ exclusion or accumulation, depending on stress conditions (Rawat *et al*., 2022; Venkataraman *et al*., 2021). Similarly, *NHX1*‐mediated Na^+^ compartmentalization and *SOS1*‐dependent Na^+^ efflux require a fine‐tuned balance of energy‐dependent proton pumps, co‐transporters and signalling pathways to efficiently enhance SST. This flexibility emphasizes the demand for customized breeding approaches that consider the precise salinity levels and environmental settings.

Innovations in modern genomics and bioinformatics tools have assisted the discovery and prioritization of complex gene networks. This ‘multi‐gene’ method not only improves the accuracy of breeding programs but also improves the probability of reaching stable and extreme SST by integrating key transporters, signalling molecules and regulatory factors into breeding strategies. However, the practical challenge persists in the number of genes that can be efficiently handled in breeding programs. It should also be highlighted that in open field conditions, plants cope with combined abiotic stresses simultaneously (known as multifactorial stress combination), which demands a gene pyramiding approach where key genes are designated and co‐expressed based on the explicit agricultural and environmental settings (Rawat *et al*., 2022; Raza *et al*., 2025b; Zandalinas *et al*., 2021b, 2024).

To design truly salinity‐smart plants, an approach that includes the regulation of ‘multiple genes’ is needed. This ‘horses for courses’ approach makes gene combinations by keeping in mind the exact salinity levels and crop requirements, leveraging advances in genomics to discover and prioritize the most appropriate gene networks, that is, ‘full gene package’ (Rawat *et al*., 2022; Raza *et al*., 2025b). In this way, future breeding programs can transform and deliver crops capable of thriving in varied combined conditions, thus improving agricultural production and sustainability.

### Genomic selection and haplotype‐based breeding: Leveraging genetic potential for stress‐smart breeding

Genomic selection (GS) is a modern breeding tool for estimating breeding value, notably for complex traits such as SST in plants (Alemu *et al*., 2024; Crossa *et al*., 2017; Kumar *et al*., 2022; Varshney *et al*., 2021c). By harnessing sequence knowledge, GS goes beyond the constraints of traditional approaches like MAS. It fast‐track genetic improvement by standardizing prediction models on genotyped and phenotyped training populations, permitting breeders to choose candidates based exclusively on genotypic data. Subsequently, this streamlines breeding plans, reducing the breeding cycle by directing complex traits early on (Alemu *et al*., 2024; Crossa *et al*., 2017; Varshney *et al*., 2017, 2021c).

For instance, a recent study including a rice reference panel of 241 accessions assessed under control and mild‐salt conditions verified substantial genotype‐by‐condition interactions for eight morphological parameters and ion mass fractions (Bartholomé *et al*., 2023). Genomic prediction methods presented predictive capabilities extending from 0.25 to 0.64 for morphological parameters and from 0.05 to 0.40 for stress response indices and ion mass fractions. Moreover, the predictive capabilities of models trained on the reference panel were validated on breeding populations of 393 accessions, with predictive capabilities extending from 0.00 to 0.66 for multi‐environment models. These findings feature the effectiveness of GS in predicting SST in rice breeding accessions, suggesting the prospective to upgrade breeding schemes for salinity conditions (Bartholomé *et al*., 2023). In maize, Singh *et al*. (2023) predicted the SST by estimating breeding values for four biomass‐associated attributes under salinity and controlled conditions. Numerous genomic prediction models were accessed, with low‐density SNPs presenting the best prediction precision for all traits. Among different models, genomic best linear unbiased prediction (GBLUP), ridge‐regression BLUP (rrBLUP) and Bayesian models surpassed others, with the average prediction precision extending from 0.46 to 0.77 across the attributes. These outcomes propose new insights for implementing and optimizing GS in breeding for SST in maize (Singh *et al*., 2023). Only a few attempts have been made to exploit the full potential of GS for accelerating salinity‐smart breeding; therefore, more efforts are required soon. Moreover, we predict that the reduced costs of genotyping and superior GS models will significantly aid in fast‐tracking the supply of superior breeding lines with SST.

Haplotypes, combinations of alleles inherited together on similar Chr, play a critical role in plant breeding for designing advanced crop varieties (Bhat *et al*., 2021; Garg, 2021). Conventional breeding relies on recombination to merge anticipated traits, but genomic regions specified by QTLs often include various candidate genes with contrasting effects. In this context, haplotype‐based breeding (HBB) utilizes genomic data to choose superior haplotypes, permitting breeders to design supreme crops *in silico* and improve future breeding programs (Bhat *et al*., 2021; Garg, 2021). Contrasting traditional breeding, which often involves lengthy cycles of crossing and selection based on phenotypic data, HBB leverages precise genomic information to recognize and select optimal haplotype combinations, significantly reducing the time and doubt associated with conventional breeding. By focusing on specific haplotype blocks associated with desirable traits (e.g. SST), HBB minimizes linkage drag and enhances the accuracy of trait introgression, which leads to faster development of superior cultivars (Bhat *et al*., 2021; Garg, 2021). HBB represents an advanced and more precise approach to breeding by design (Peleman and Van der Voort, 2003). For example, while traditional methods might require multiple generations to achieve the desired trait combinations, HBB can fast‐track this process by directly selecting the most beneficial haplotypes from the outset and bypassing the uncertainty and labor‐intensive work of trial and error typically associated with traditional breeding (Bhat *et al*., 2021; Garg, 2021).

Earlier, Patil *et al*. (2016) focused on improving SST in soybean through haplotype analysis and MAS. With the help of high‐quality whole‐genome resequencing of diverse soybean lines, three major structural and allelic variants in the *GmCHX1* gene were identified. Through haplotype analysis and pedigree tracking, SNP markers were validated and showed a strong relationship with salinity treatment phenotypes. These markers accurately distinguished salinity‐tolerant/sensitive genotypes and distinct structural variants, assisting in designing salinity‐smart soybean cultivars (Patil *et al*., 2016). Another soybean study identified new haplotypes of the SST‐related gene *GmSALT3* and designed molecular markers for choosing salinity‐tolerant and ‐sensitive accessions (Lee *et al*., 2018). Genomic variants in the *GmSALT3* coding region of 216 accessions from Korea, China and Japan were explored, and 40 diverse haplotypes containing three known ones were discovered. Through SST tests, these haplotypes were grouped into salinity‐tolerant and salinity‐sensitive groups, and the quantitative expression evaluation displayed higher expression patterns of *GmSALT3* in salinity‐tolerant haplotypes. Moreover, molecular markers were developed and employed, representing 98.8% precision in discovering soybean accession phenotypes for SST (Lee *et al*., 2018). Through inclusive genomic analysis and marker designing, both studies presented significant insights into discovering and picking salinity‐tolerant genotypes for designing salinity‐smart future soybean cultivars.

The genetic basis of SST was investigated in wheat using a panel of 307 accessions, including local landraces and exotic cultivars, via GWAS (Yu *et al*., 2020). The results of marker‐ and pedigree‐based kinship analysis discovered that helpful haplotypes were hosted in some exotic cultivars and early Chinese landraces. Nevertheless, improvements in SST in advanced breeding were less pronounced than yield improvements. Breeders must focus on local landraces with high tolerance to increase SST and rare promising alleles yet to be employed in breeding efforts (Yu *et al*., 2020). More recently, Pasam *et al*. (2023) explored 580 wheat accessions for SST using digital phenotyping, uncovering attributes like digital shoot growth rate as markers of SST. Through a haplotype‐based GWAS, 95 QTLs for SST were detected, including 54 novel ones. This study also focuses on the extensive nature of SST across diverse germplasm, proposing small‐effect genetic variations that influence tolerance levels. Hence, it should be noted that accessions with altered tolerance mechanisms recommend promising further genetic studies and breeding efforts to improve SST in wheat (Pasam *et al*., 2023).

Ravikiran *et al*. (2018) assessed 192 rice genotypes for seedling‐stage SST using a combination of morpho‐physiological and molecular markers. Thirteen SSR markers connected with the *Saltol* region on Chr1 were diagnosed, which resulted in two efficient markers (RM 493 and RM 10793) for genotype distinction. Haplotype analysis of *Saltol*‐related markers identified potential novel genomic regions regulating SST, and two genotypes (CST 7–1 and Arvattelu) emerged as promising candidates for salinity‐smart rice breeding (Ravikiran *et al*., 2018). In another rice study, haplotype analysis showed six InDel markers correlated with SST traits, delivering helpful tools for molecular discovery of salinity‐smart rice accessions (Yang *et al*., 2023a). GWAS‐based haplotype analysis of newly identified 35 genes showed that four of them, including *LOC_Os12g34450*, displayed extensively unique haplotypes allied with SST (Xu *et al*., 2023a). Notably, *LOC_Os12g34450* showed two haplotypes differentiated by non‐synonymous mutations in the exon region. Furthermore, haplotype analysis of other genes detected SNPs in promoter and exon regions. Overall, the combination of GWAS, haplotype analysis, qRT‐PCR and sequence examination discovered *LOC_Os12g34450* gene as a promising target for designing salinity‐smart rice (Xu *et al*., 2023a). More recently, Liu *et al*. (2024) carried out GWAS‐based haplotype analysis of 541 rice accessions, discovering a key gene *OsTMF* harbouring four haplotypes (Hap1, Hap2, Hap3 and Hap4). Of these, rice varieties carrying *OsTMF‐Hap2* exhibited superior SST during seed germination, providing new insights for fast‐tracking stress‐smart rice breeding (Liu *et al*., 2024).

In a nutshell, integrating HBB with advanced genomic methods such as haplotype analysis and MAS demonstrates the potential for improving SST in major crops, for example, soybean, wheat, rice and others. These findings highlight the value of discovering novel haplotypes associated with SST‐related genes, facilitating the design of salinity‐smart cultivars. Future attempts should investigate diverse germplasm to expose further rare alleles and genomic regions underlying SST while improving genomic tools for accurately selecting superior haplotypes. Furthermore, accepting a strategy ‘haplo‐GS’, proposed by Varshney *et al*. (2021a), which combines GS with superior haplotypes and its integration with speed breeding (a controlled environment‐based method to accelerate plant growth and reproductive cycles) (Watson *et al*., 2018), offers an influential method to quickly adapt crop varieties with higher performance across manifold adaptive parameters and can also fast‐track the development of stress‐smart crop varieties.

### Pan‐genomics: An emerging method for fast‐forward breeding

If SST implies multiple genes working together, it could be compromised if even a single important component is absent. Imagining that our modern crops retain most of the vital elements but lack some, it may be feasible to discover these missing pieces. Nevertheless, the challenge remains in discovering these absent elements. Could pan‐genomics be the solution to discover these missing components? With recent advancements in plant genome sequencing technologies, pan‐genomics extends assurance for finding these missing genetic pieces with its inclusive analysis of large genomic variations within species. By comparing the genomes of salinity‐tolerant and sensitive varieties, pan‐genomics can discover the critical genes and pathways that contribute to SST, presenting new insights for breeding programs that enhance SST in crops. The pan‐genome covers core genes present across all individuals and unpredictable genes missing in some, suggesting a broad view of genetic diversity (Bayer *et al*., 2020; Danilevicz *et al*., 2020; Lei *et al*., 2021; Mishra *et al*., 2024; Raza *et al*., 2023b; Shi *et al*., 2022; Wang *et al*., 2023; Zanini *et al*., 2022).

Likewise, the emergence of super pan‐genomics (or super‐pangenome) is an advanced extension of conventional pan‐genomics that integrates high‐resolution genomic data from hundreds to thousands of diverse accessions, including CWRs and landraces, to capture a complete picture of species/genus‐wide genetic diversity (Khan *et al*., 2020; Raza *et al*., 2023a). This approach leverages the genomic landscape within cultivated gene pools, portraying extensive variations across accessions from accessible species, as explained in an authoritative review by Khan *et al*. (2020). Unlike traditional pan‐genomics, which typically focuses on core (shared) and dispensable (variable) genes within a limited set of reference genomes (Bayer *et al*., 2020; Danilevicz *et al*., 2020; Lei *et al*., 2021; Shi *et al*., 2022; Zanini *et al*., 2022), super pan‐genomes employ graph‐based and telomere‐to‐telomere assemblies and long‐read sequencing to discover rare alleles, structural variants and presence–absence variations at a record scale, thus delivering more profound insights into adaptive traits and evolutionary mechanisms (Garg *et al*., 2024; Khan *et al*., 2020; Raza *et al*., 2023a). This paradigm shift confronts the capability of single reference genomes and highlights the significance of pan‐genomic methods in identifying genetic diversity. Particularly, pan‐genomes enable the discovery of huge structural variants that play critical roles in phenotypic diversity and adaptation (Bayer *et al*., 2020; Danilevicz *et al*., 2020; Khan *et al*., 2020; Lei *et al*., 2021; Mishra *et al*., 2024; Raza *et al*., 2023a, 2023b; Shi *et al*., 2022). Plant pan‐genomics proposes smart opportunities for designing improved cultivars by leveraging the affordability of sequencing technologies.

During the last 3 years, there has been a wave for applying plant pan‐genomics (i.e. the development of pan‐genomes and super pan‐genomes) that yielded 921 genomes via pan‐genome projects (Xie *et al*., 2024) across multiple plant species such as *Arabidopsis*, soybean, tomato, wheat, rice, maize, barley, cotton, sorghum, potato, citrus, rapeseed, radish, peas, Chinese cabbage, cucumber, mungbean, pearl millet, grapes, mangrove, Populus, sesame, sunflower, walnut, peach, etc. (Bayer *et al*., 2020; Danilevicz *et al*., 2020; Lei *et al*., 2021; Raza *et al*., 2023a, 2023b; Shi *et al*., 2022; Wang *et al*., 2023; Xie *et al*., 2024; Zanini *et al*., 2022), delivering visions into the improvement of diverse traits such as agronomic, biotic and abiotic stress tolerance by discovering new genes that are not present in the reference genomes. Therefore, it should be noted that these pan‐genomics resources have massive potential in transforming plant breeding for increasing SST by discovering novel genetic variations correlated with SST that traditional methods might miss.

For instance, a pan‐genome analysis of radish has identified a new gene (*RsGA2ox7*) possessing diverse stress‐responsive *cis*‐elements (Zhang *et al*., 2021). Under stress conditions, its expression level was notably upregulated in both mutant and WT plants, suggesting its vital role against waterlogging, drought, cold and salinity stresses (Zhang *et al*., 2021). In the future, this gene could potentially be manipulated to design stress‐smart radish cultivars. Using pan‐GWAS analysis, the super pan‐genome of North American wild grape species has identified numerous loci associated with SST in a natural population (Cochetel *et al*., 2023). Of these, one candidate region (*VITVgdSC2_v1.0.hap1.chr08.ver1.0.g144890.p01*) was discovered, displaying homology with *A. thaliana* cation/H^+^ exchanger *AtCHX20* (*AT3G53720*). The assumed function of *AtCHX20* was further verified by the presence of two important InterPro domains: the Cation/H ^+^ exchanger (IPR006153) and the Sodium/solute symporter superfamily (IPR038770). Furthermore, the gene upstream of the significant SNP also showed homology with *AtCHX20*. These outcomes highlight the power of super pan‐genome for discovering the base‐level associations associated with SST (Cochetel *et al*., 2023). Recent studies have increasingly leveraged super pan‐genomics to discover the genetic basis of SST. For example, Wei *et al*. (2024) used the super pan‐genome map of rice and discovered 22 345 and 27 610 eQTLs correlated with the expression of 7 787 and 9 361 eGenes under salinity and control conditions. By integrating eQTL and GWAS analysis, a potential gene (*STG5*, a major locus *qSTS5*) has been identified as playing a key role in SST. Notably, *STG5* was found to control Na^+^/K^+^ homeostasis by promptly changing the transcription of various *OsHKT* genes. This exciting study presents the genetic landscapes of gene expression under salinity stress and opens new windows for exploring SST‐related networks and salinity‐smart breeding (Wei *et al*., 2024).

In a subsequent experiment using the rice super pan‐genome, Cui *et al*. (2024b) discovered 2427 and 2898 *cis*‐PAV‐eGenes under normal and salinity conditions, respectively. They focused on 22 transcription factors overlapping with DEGs and dynamic PAV‐eGenes under salinity stress, finding that 12 had important associations with SST. Of these, *OsMADS56* (*GL10*) turned out to be a key element for SST in rice. Notably, one PAV (Chr10_20,863 489) interrupted its expression, triggering high salinity sensitivity. Near‐isogenic lines (NILs) confirmed that NIL‐*GL10* had higher survival rates under salinity stress compared to NIL‐*gl10*. Furthermore, the overexpression of *OsMADS56* enhanced SST by reducing ROS accumulation. This gene also induces grain size, thermotolerance and photoperiodic flowering, becoming a beneficial target for developing improved rice cultivars (Cui *et al*., 2024b).

These pan‐genomics studies suggest insights into stress response pathways and deliver potential targets for improving SST in plants. By identifying genomic regions under positive selection in salinity‐smart populations (e.g. halophytes), pan‐genomics facilitates the integration of SST‐associated genes into breeding programs through GAB or genome editing techniques. Future research directions should include discovering the functional significance of genetic variations, translating their underlying mechanisms and employing them to design salinity‐smart crop varieties through precision breeding methods. Moreover, diverse germplasm collections from separate geographic origins, including cultivated, landraces and CWR, deliver new resources for pan‐genomics analysis. The data on structural variants, loci and core genes obtained from these collections could be further applied in various fast‐forward breeding methods: transgenomics, genome editing and GAB/GS (Mishra *et al*., 2024; Raza *et al*., 2023a, 2023b; Varshney *et al*., 2021b). These integrated methods can advance various crop traits, including SST, and contribute to sustainable food production in the face of changing climate.

### Single‐cell genomics: A new wave for stress‐smart breeding

Single‐cell genomics (sc‐genomics, mainly sc‐RNA‐seq) technologies have transformed research by proposing exceptional sensitivity and throughput, helping the profiling of cell‐specific genomic features (Nolan and Shahan, 2023; Stuart and Satija, 2019). This approach delivers an extraordinary resolution of gene expression, regulatory networks and phenotypic features at cellular levels, discovering hidden cellular heterogeneity and empowering the documentation of stress‐responsive pathways that bulk tissue investigations frequently overlook. Though sc‐genomics has been applied at different developmental stages and for understanding a few abiotic stresses (Cuperus, 2022; Luo *et al*., 2020; Nolan and Shahan, 2023; Zhu *et al*., 2022b), the studies are still minimal under salinity stress.

Sc‐RNA‐seq has proven its utility in uncovering cell‐type‐specific responses under salinity stress. Dinneny *et al*. (2008) verified that salinity and iron deficiency prompted highly cell‐type‐ and developmental‐stage‐specific transcriptional responses in *Arabidopsis* root cells. Recent improvements have extended this method to diverse plant systems. For instance, Liu *et al*. (2022b) used sc‐RNA‐seq to explore the expression levels of PIFs in leaf epidermal and guard cells under drought and salinity stresses, suggesting their role in regulating cell morphology and stress tolerance. Likewise, Li *et al*. (2024c) built a sc‐transcriptome atlas of salinity‐stressed cotton roots, detecting 10 distinct cell types and tracking changes in cell populations. They highlighted stress‐specific roles of outer epidermal and inner endodermal cells and confirmed the functional contribution of candidate genes ‘*GaGH3.6*’ in maintaining redox homeostasis and improving SST in cotton (Li *et al*., 2024c). These reports highlight the power of sc‐genomics to identify stress‐tolerant cellular states and transcriptional networks triggering stress responses.

Recent innovations in spatial transcriptomics have introduced a new method, ‘PHYTOMap’, a transgene‐free multiplexed FISH technology, assisting high‐resolution sc‐spatial analysis of gene expression in whole‐mount plant tissues (Nobori *et al*., 2023). This approach validated marker genes identified in sc‐RNA‐seq data in *Arabidopsis* roots, precisely mapping transcriptional activity across cell types. Such accessible tools can notably enhance our understanding of cellular responses against salinity stress and support breeding strategies in non‐transgenic species (Nobori *et al*., 2023).

In contrast to conventional whole‐plant assessments, sc‐genomics or sc‐RNA‐seq guides the examination of cellular responses at an extraordinary resolution, presenting perceptions of how individual cells within various tissues respond to salinity stress (Barkla *et al*., 2018; Carden *et al*., 2003; Wang *et al*., 2021; Zhao *et al*., 2024). This granularity is vital for discovering key regulatory networks and cell‐specific responses that promote overall plant tolerance and health. Sc‐genomics, together with sc‐based phenotyping, can provide new insights for understanding how plants respond to stresses like salinity, which is crucial for designing stress‐smart plants. Moreover, sc‐RNA‐seq offers unique insights into how plant cells display stress‐specific responses regulated by their location and purpose, emphasizing genes, regulatory elements and pathways essential for stress adaptation.

Salinity stress induces significant changes in membrane dynamics, and this phenomenon was evaluated in epidermal bladder cells of *Mesembryanthemum crystallinum*, as a sc‐model (Barkla *et al*., 2018). Salinity triggers alterations in lipid composition, with increased phospholipids and decreased triacylglycerols, implicating lipid metabolism against stress (Barkla *et al*., 2018). Additionally, sc‐C_4_ photosynthesis in *Bienertia sinuspersici* highlights the unique adaptations to salinity environments (Leisner *et al*., 2010). The results illuminated the complex interplay between salinity, growth, and photosynthesis that provides insights into the functioning of the sc‐C_4_ system (Leisner *et al*., 2010).

In rice, sc‐genomics examined cell type‐specific responses to low nitrogen, high salinity and iron deficiency stresses (Wang *et al*., 2021). High salinity treatment reduced the mesophyll cell population and chlorophyll abundance per cell. Whereas transcriptomic data exposed a delay in mesophyll cell development under high salinity, with suppressed genes associated with proliferation and photosynthesis (Wang *et al*., 2021). Recently, sc‐RNAseq of recretohalophyte sea lavender (*Limonium bicolor*) during salinity gland formation showed 19 distinct cell clusters and confirmed the role of cytokinin in the differentiation activity. They also discovered specific differentiation trajectories of salinity gland formation‐related sub‐clusters and the contribution of *TRIPTYCHON* and *Lb7G34824* proteins in regulating cytokinin homeostasis during salinity gland formation (Zhao *et al*., 2024). However, most existing studies highlight the physiological traits of stress responses in specific cell types. The integration of sc‐genomics and sc‐phenotyping bridges the gap between cellular resolution and functional genomics. This integration can transform breeding efforts by providing a deeper understanding of how individual cells respond and adapt to salinity stress.

Sc‐genomics coupled with sc‐based phenotyping are exciting opportunities to investigate the complex mechanisms driving plant responses to salinity stress (see Section “*Drilling down from whole‐plant to single‐cell/tissue: Overcoming phenotyping bottlenecks*” for more arguments on sc‐based phenotyping). Nonetheless, despite advancements, the application of sc‐genomics in understanding salinity responses is still limited, mostly due to the lack of a bridge between genotype and phenotype at the cellular level. In this framework, integrating sc‐genomics with HTP can substantially increase our understanding and capability to breed salinity‐smart crops. Therefore, more efforts are required to apply sc‐genomics to discover additional stress‐responsive pathways and assist the development of stress‐smart plants. Integrating spatial transcriptomics with sc‐genomics will deliver new insights into tissue‐/cell‐specific gene expression trends and their influence on plant development under salinity. Moreover, we also need to focus on developing the spatiotemporal dynamics of cellular responses to salinity and understanding the maintenance of cell identity across conditions. This will specify an understanding of developmental plasticity and stress adaptation mechanisms that are required for targeted crop breeding strategies under stressful conditions.

### Epigenomics: A key frontier for harnessing salinity tolerance

Epigenomics or epigenetics is a functional genomic tool with great potential to enhance plant adaptation and tolerance to diverse stresses, including salinity (Cao and Chen, 2024; Saeed *et al*., 2022; Singroha *et al*., 2022). Across complex modifications in DNA and histone proteins, it regulates stress memory and gene expression deprived of altering the DNA sequence (Cao and Chen, 2024; Saeed *et al*., 2022). Understanding this dynamic interaction discovers new ways of designing salinity‐smart cultivars.

Plant DNA methylation occurs with N6‐methyladenine (6 mA) or 5‐methylcytosine (5 mC) (Gallego‐Bartolomé, 2020). In the context of salinity, 6 mA remains less well‐studied, and most reports focus on the role of 5mC in regulating plant responses to salinity conditions. For instance, increased DNA methylation was examined in barley leaves rather than in roots, and it was claimed that salinity‐induced methylation is organ‐specific (Konate *et al*., 2018). In soybean roots, 61.2% of CGs, 39.7% of CHG, and 3.2% of CHHs were methylated under salinity, representing significantly lower methylation than the control environment (Chen *et al*., 2019). This pattern suggests that lower methylation levels may be linked to gene activation and enhanced SST. Another study by Wang *et al*. (2020) observed improved methylation at CHH and CHG context in Miniature Inverted Repeat Transposable Elements in *OsHKT1;5* gene under salinity stress. Furthermore, the critical participation of methylation in regulating *OsHKT1;5* was also noticed, contributing to SST in rice (Wang *et al*., 2020). Due to its heritable character, salinity‐induced DNA methylation modifications have the potential to be stable and be transferred to future generations. This idea gives the green light to stress‐induced methylation shifts to act as a ‘form of memory’, which help plant or its offspring to better adapt to the salinity stress upon re‐exposure (Cao and Chen, 2024).

Epigenetic diversity can play an important role in the adaptive evolution and breeding of plants in stressful conditions (Cao and Chen, 2024). For instance, field pennycress (*Thlaspi arvense*) exhibited increased epigenetic diversity under salinity stress, and this effect was partly transferred to at least two generations of offspring in non‐stressed environments (Geng *et al*., 2020). Likewise, rice plants showed transgenerational plasticity, with the progeny of salinity‐stressed parents exhibiting improved SST (Aycan *et al*., 2024). In a domesticated halophyte (*Puccinellia tenuiflora*), growing the plant under non‐saline conditions led to alterations in DNA methylation, which in turn altered salinity tolerance genes. These alterations could assist in the swift domestication of this species (Li *et al*., 2021a). Maternal effects in wheat highlight the impact of the maternal genotype on SST in offspring, independent of nuclear genomic factors or DNA methylation processes (Aycan *et al*., 2021). These examples feature the ability to harness transgenerational epigenetic modifications to design salinity‐smart cultivars.

It should be mentioned that some epigenetic modifications, including DNA methylation, may change during mitotic and meiotic cell division, restricting their success in successive generations (Kawakatsu and Ecker, 2019). This uncertainty requires more research to discover stable epigenetic modifications that can be preserved across generations or to develop methods that guide the discovery or tracking of these modifications in progeny (Cao and Chen, 2024). Beyond DNA methylation, histone modifications further regulate stress‐responsive gene expression through chromatin remodelling, shaping plant tolerance under salinity stress.

Histones are proteins containing lysine and arginine residues that determine the basis of nucleosome chromatin organization (Bajpai *et al*., 2024). In salt‐stressed maize roots, key cell wall‐associated genes, such as *ZmEXPANSIN* B2 and *ZmXYLOGLUCAN* endotransglucosylase/hydrolase1, display upregulation credited to improved *H3K9* acetylation at their promoter and coding regions, driven by augmented mRNA expression of *histone acetyltransferase* (*HAT*) genes like *ZmHATB* and *ZmGCN5* (Li *et al*., 2014). These discoveries were corroborated in Arabidopsis, where the involvement of *HAT* genes in regulating SST has been presented, with the *GCN5* mutant displaying higher Na^+^ uptake and accumulation than WT plants, damaging its growth under salinity (Zheng *et al*., 2019). Furthermore, *GCN5* interacts with cell wall synthesis‐related genes, including *CTL1* and *MYB54*, that emphasize its role as a conserved epigenetic regulator in response to salinity stress (Zheng *et al*., 2019).

Some *HAT* genes exhibit increased *H4K5* acetylation under salinity stress, and certain histone deacetylases respond negatively to SST. For example, Cheng *et al*. (2018) investigated the role of *IDS1* against SST in rice. By employing ChIP sequencing and assays, the authors discovered that *IDS1* directly interacts with GCC‐box‐holding motifs in the promoters of key abiotic‐receptive genes like *LEA1* and *SOS1*. Moreover, they also found that *IDS1* physically interacts with *topless‐related 1* and *HDA1*, leading to the repression of *LEA1* and *SOS1* expression. This epigenetic mechanism shows how the *IDS1* gene regulates salinity stress signalling and enhances SST in rice (Cheng *et al*., 2018). In wheat, a histone acetyltransferase gene (*TaHAG1*) was vital for boosting SST (Zheng *et al*., 2021). The expression of *TaHAG1* increases during salinity stress, influencing ROS production and targeting specific genes for hydrogen peroxide production. This epigenetic mechanism develops wheat flexibility and guides a potential approach for salinity‐smart breeding (Zheng *et al*., 2021).

Identifying key regulators like histone acetyltransferases shows the possible use of epigenetic modulators in stress‐smart breeding programs. Yet, to fully harness this potential, further investigations are needed to explore epigenomic mechanisms in major food crops and advance strategies to confirm the stability of advantageous epigenetic traits throughout generations (Cao and Chen, 2024).

### Transgenomics: A paradigm shift from ‘one‐to‐another’ for improving salinity tolerance

Transgenomics, also referred to as transgenic breeding, is an extensively used targeted gene‐based method that offers significant perceptions into gene regulation, exceptionally under stress conditions (Correa *et al*., 2012; Singh *et al*., 2021). It implicates the transfer of foreign genes encoding important agronomic traits, for example, SST, from one organism to a target host plant ‘one‐to‐another’. With the help of transgenomics, various novel phenotypes have been developed, improving our understanding of stress‐responsive genes in various crops (Table 3). By introducing specific genes (but how many genes are enough? See Section “*Prioritizing gene networks for salinity tolerance: A ‘horses for courses’ approach*” for more arguments on ‘horses for courses’ approach) or genetic elements into crop plants, transgenomics facilitates accurate manipulation of gene expression to develop stress‐smart phenotypes (Raza *et al*., 2025b). Additionally, the transgenomics method complements GS and other breeding approaches by directly integrating known stress tolerance genes into breeding populations, consequently fast‐tracking the designing of stress‐smart crop varieties to meet future food demands.

**Table 3:** Transgenic plants with improved salinity tolerance developed by the application of transgenomics

Source plant	Botanical name	Stress condition	Targeted gene	Key advancement	References
From major crop to model organism *Arabidopsis thaliana* (a host plant)					
Barrelclover	*Medicago truncatula*	200 mM NaCl; 2, 4, 8, 12 and 24 h	*MtDof32*	Enhanced tolerance for osmotic and salinity stresses Increased rosette number Enlarged flower and leaf organs	Guo *et al*. (2021a)
Indian Winter cherry	*Withania somnifera*	150 mM NaCl; 2, 4, 8, 16, and 24 h	*SGT* gene (*WssgtL3.1*)	Higher germination rate Decreased H_2_O_2_ and MDA accumulation Defensive role against salinity stress with firm membrane integrity	Mishra *et al*. (2021)
Sweet potato	*Ipomoea batatas*	100 mM NaCl; 7 and 14 d	*IbATL38*	Reduced H_2_O_2_ contents Upregulates genes responsible for ROS scavenging systems	Du *et al*. (2021)
Maize	*Zea mays*	100 and 150 mM NaCl; 3, 7 and 14 d	*ZmEREB20*	Increased survival rate Enhanced ROS scavenging systems Maintained ion homeostasis Improved root hair growth	Fu *et al*. (2021)
Rice	*Oryza sativa*	100, and 200 mM NaCl; 48 h	*OsMADS57*	Improved germination rates and growth status Longer root length Reduced ROS accumulation and MDA content	Wu *et al*. (2021)
Wheat	*Triticum aestivum*	50 mM NaCl; 7 d	*TaHSP17.6*	Increased number of lateral roots Higher accumulation of proline Boosted the activity of the POD enzyme	Qin *et al*. (2022)
Wheat	*T. aestivum*	200 mM NaCl; 1, 3, 6, 9, 12 and 24 h	*TaTIP4;1*	Improved seed germination and seedling growth Improved CAT and SOD activities proline accumulation Increased water contents, decreased leaf water loss, and levels of Na^+^, Na^+^/K^+^, MDA and H_2_O_2_	Wang *et al*. (2022)
Soybean	*Glycine max*	70 mM NaCl and 50 mM NaHCO3; 1, 3, 6, 12, 24, and 48 h	*GmSNF1*	Increased the expression of the *CAT* biosynthesis genes Increased the expression of stress‐related genes Enhanced CAT activity	Lu *et al*. (2023)
Soybean	*G. max*	120 mM NaCl; 3, 6, 12, and 24 h	*GsWRKY23*	Improved growth appearance, plant height and fresh weight Improved leaf Chl and RWC contents Reduced relative electrolytic leakage and MDA contents	Sun *et al*. (2023)
Wheat	*T. aestivum*	200 mM NaCl; 1, 2, 4, 6, 12 and 24 h	*TaF5H1*	Improved biomass yield and S‐lignin content	Jia *et al*. (2023)
Wheat	*T. aestivum*	200 mM NaCl; 1, 3, 6, 12, and 24 h	*TaWRKY17*	Increased the activities of SOD, POD and CAT enzymes Decreased H_2_O_2_ and MDA accumulation Increased expression levels of ABA and ROS‐related genes	Yu *et al*. (2023)
Wheat	*T. aestivum*	1 M NaCl; 24 h	*TaGPX1‐D*	Improved germination rates and antioxidant enzyme activities Improved Chl, carotenoid, proline, and RWC contents Reduced H_2_O_2_ and MDA levels	Tyagi *et al*. (2023)
Rice	*O. sativa*	75 mM NaCl; 8 d	*OsOBP4*	Enhanced root growth Improved expression of *AGL7* for floral meristem induction and light reaction protection	Jakada *et al*. (2024)
Rice	*O. sativa*	250 mM NaCl; 1 and 4 d	*OsFes1A*	Improved germination rate Improved phytosterols and Chl content Reduced MDA content Improved the activity of POD	Xu *et al*. (2024)
Beta grape	*Vitis vinifera*	100 mM NaCl; 1, 2, 4, 8, 12, and 24 h	*VhMYB15*	Reduced yellowing and wilting of leaves Increased survival rate Improved the expression levels of stress‐associated genes	Han *et al*. (2024)
Maize	*Z. mays*	300 mM NaCl; 3, 6, 12, and 24 h	*ZmCBL8‐1*	Reduced H_2_O_2_ and MDA levels Increased the expression levels of antioxidant‐associated and Na^+^/H^+^ antiporter genes	Wang *et al*. (2024)
From *Arabidopsis thaliana* to major crop (a host plant)					
Indian mustard	*Brassica juncea*	200 mM NaCl; 10 d	*AtApx1*	Decreased H_2_O_2_ levels Lesser membrane damage Enhanced proline accumulation and Chl stability index Improved antioxidative enzyme activities	Saxena *et al*. (2020)
Key lime	*Citrus aurantifolia*	100 mM NaCl; 24 h	*AtCBF3*	Increased Chl content Less accumulation of ROS	Romero‐Romero *et al*. (2020)
Rice	*O. sativa*	100 mM NaCl; 35 d	*AtDREB1A*	Enhanced survival rate Enhanced germination and retention of Chl Higher RWC% and decreased leaf temperature	Muthurajan *et al*. (2021)
Wheat	*T. aestivum*	150 and 200 mmol L^−1^ NaCl; 8 and 30 d	*AtOAT*	Increased the expression of the proline biosynthesis‐related genes (*TaOAT*, *TaP5CS*, and *TaP5CR*) Reduced the expression of proline catabolism‐related gene (*TaP5CDH*) Increased the expression of genes involved in ornithine pathway (*Orn‐OAT‐P5C/GSA‐P5CR‐Pro*) and glutamate pathway (*Glu‐P5CS‐P5C/GSA‐P5CR‐Pro*)	Anwar *et al*. (2021)
Wheat	*T. aestivum*	50, 100, and 200 mM NaCl; 1 week intervals	*AtCIPK16*	Helped to retain more K^+^ ions Positive elicitor in sequestering Na^+^ ions	Imtiaz *et al*. (2023)
Within the same crop as host and source plant					
Rice	*O. sativa*	200 or 300 mM NaCl; 7 d	*OsACBP4*	Regulated lipid metabolism Increased the expression levels of genes encoding acyl‐CoA synthase, contributing to fatty acid elongation	Guo *et al*. (2021b)
Rice	*O. sativa*	100, and 200 mM NaCl; 48 h	*OsMADS57*	Improved germination rates and growth status Longer root length Reduced MDA content and ROS accumulation Improved oxidative tolerance Increased the activities of SOD and POD enzymes Improved expression level of stress‐related genes	Wu *et al*. (2021)
Maize	*Z. mays*	200 mM NaCl; 3, 6, 9, 12, 24, and 36 h	*ZmBSK1*	Reduced H_2_O_2_ and O^2−^ accumulation, and MDA content Increased antioxidant enzyme activities and proline content Increased the expression levels of ROS scavenging‐ and proline biosynthesis‐associated genes	Liu *et al.* (2022a)
Maize	*Z. mays*	150 mM NaCl; 12 h and 14 d	*ZmSRG7*	Improved germination rate and root length Increased Na^+^ concentration and Na^+^: K^+^ ratio Improved antioxidant enzymes and proline contents Reduced MDA, H_2_O_2_ and O^2−^ contents	Wei *et al*. (2022)
Soybean	*G. max*	150, 200 or 250 mmol L^‐1^ NaCl; 6, 12, 24, and 48 h	*TCP9‐like*	Lower Na^+^ and higher K^+^ accumulations Higher K^+^/Na^+^ ratio Increased the expression levels of key genes involved in e K^+^/Na^+^ homeostasis pathway	Zhang *et al.* (2023e)
Maize	*Z. mays*	100 or 150 mM NaCl solution; every 2 weeks	*ZmSTG1*	Increased plant growth vigor Improved photosystem II activity Modulated the lipid composition	Mei *et al*. (2023)
Rice	*O. sativa*	150 mM NaCl; 1 d	*OsERF106MZ*	Increased the length of the primary root Roots displayed reduced sensitivity to ABA‐mediated root growth inhibition	Chen *et al.* (2023a)
Rice	*O. sativa*	200 mM NaCl; 5 d	*OsDB10*	Improved seed germination, growth survival rate, and grain yield Reduced necrosis with higher Chl content Accumulated lower Na^+^ and higher K^+^ ions Improved oxidative stress by ROS scavenging and by antioxidant defense system	Banu *et al*. (2023)
Rice	*O. sativa*	200 mM NaCl; 6 d	*OsDUF6*	Increased the activities of SOD, POD, CAT, and phenylalanine ammonia‐lyase Positively regulated Na^+^ transport Improved the expression level of growth‐ and stress‐related genes	Ma *et al*. (2024)
Rice	*O. sativa*	200 mM NaCl; 24 and 48 h	*OsRR26*	Diverse cellular ROS compartmentalization Improved Chl retention Improved the accumulation of soluble sugars, K^+^ content, and proline	Nongpiur *et al*. (2024)
Wheat	*T. aestivum*	200 mM NaCl; 3 weeks	*TaHKT9‐B*	Decreased the K^+^ accumulation and thus controls K^+^/Na^+^ homeostasis *TaARF4* acts as a negative regulator of salinity tolerance	Du *et al*. (2024)

Abbreviations: ABA, abscisic acid; CAT, catalase; Chl, chlorophyll; H_2_O_2_, hydrogen peroxide; K^+^, potassium ion; MDA, malonaldehyde; Na^+^, sodium ion; NaCl, sodium chloride; O_2_^_^, superoxide anion; POD, peroxidase; RWC, relative water contents; SOD, superoxide dismutase.

As a model organism, *A. thaliana* has played a vital role in understanding SST mechanisms, and genes discovered using Arabidopsis have been translated to major crop plants. Introducing diverse SST‐responsive genes into crop plants through transgenomics has enhanced SST tolerance in transgenic plants (Table 3). For example, *AtApx1* confers SST in Indian mustard (Saxena *et al*., 2020), *AtCBF3* in Mexican lemon (Romero‐Romero *et al*., 2020), *AtCIPK16* (Imtiaz *et al*., 2023) and *AtOAT* (Anwar *et al*., 2021) in transgenic wheat, *AtICE1* (Verma *et al*., 2020), *AtDREB1A* (Muthurajan *et al*., 2021) and *AtAVP1* (Chairunisa *et al*., 2023) in transgenic rice and *AtSOS3* in *Petunia* (Madadi *et al*., 2022).

Likewise, genes from several higher plants have been transferred and overexpressed successfully into Arabidopsis, and the resultant transgenic plants display enhanced SST (Table 3). For instance, *GmSNF1* from soybean improves SST in transgenic arabidopsis (Lu *et al*., 2023), *TaF5H1* (Jia *et al*., 2023), *TaWRKY17* (Yu *et al*., 2023) and *TaGPX1‐D* from wheat (Tyagi *et al*., 2023), *OsOBP4* (Jakada *et al*., 2024), *OsMS* (Thanabut *et al*., 2023), *OsBTBZ1* (Saputro *et al*., 2023) from rice, *AhMYB30* from peanut (Chen *et al*., 2023b), *GsWRKY23* from soybean (Sun *et al*., 2023) and *ZmTIFY16* (Zhang *et al*., 2023a) and *ZmNAC2* from maize (Chen *et al*., 2023c).

Likewise, transgenomics has been successfully applied to various major field crops (Table 3). For instance, transgenic rice plants expressing *OsDB10* displayed faster seed germination and seedling growth, decreased necrosis, elevated chlorophyll, increased survival rate and improved grain yield against salinity stress (Banu *et al*., 2023). Transgenic rice also showed boosted tolerance to salinity‐induced oxidative stress by ROS scavenging and improved antioxidant defense systems (Banu *et al*., 2023). Transgenic wheat overexpressing *TabZIP60* showed improved SST with better growth levels, elevated soluble sugar, lower MDA contents and high ABA content. Overall, *TabZIP60* is a key player in regulating ABA‐mediated SST by interacting with *TaCDPK30* in transgenic wheat (Zhang *et al*., 2023b). Overexpression of *OsWRKY28* confers SST in rice by immediately attaching to the *OsDREB1B* promoter and enhancing its transcriptional action and adversely controls ABA‐mediated seedling emergence in transgenic rice (Zhang *et al*., 2023c). Another rice study directed that the overexpression of *OsERF106MZ* played a vital role in preserving root growth for source uptake against salinity by improving ABA‐mediated root growth inhibition (Chen *et al*., 2023a). Overexpression of *ZmSTG1* improved plant growth vigor and SST by improving photosynthetic activity in transgenic maize plants (Mei *et al*., 2023). In soybean, the overexpression of *TCP9‐like* increased the SST by lower Na^+^ and higher K^+^ accumulation (Zhang *et al*., 2023e). These examples highlight the scope of generating transgenic major crops to cope with salinity stress and contribute to food security.

Transgenomics, as a transformative tool, sheds light on stress‐related gene regulation for designing stress‐smart crop plants. GAB can fast‐track this procedure by discovering new genes and regulatory systems associated with SST, thus finding targets for transgenomics interventions. By combining GAB tools with transgenomics, scientists can more efficiently design and authorize genetic adjustments for increased crop tolerance. Nevertheless, transitioning from lab findings to field application remains challenging, specifically in major food crops. Bridging this gap is necessary to breed stress‐smart crops qualified for sustaining high yields under salinity conditions. Notwithstanding its vast potential, regulatory obstacles and restricted commercial integration impede the extensive adoption of transgenomics technologies, hindering their full application in climate‐smart agriculture.

## Integration of high‐throughput phenotyping and artificial intelligence within the context of stress‐smart GAB

**Figure 4:**
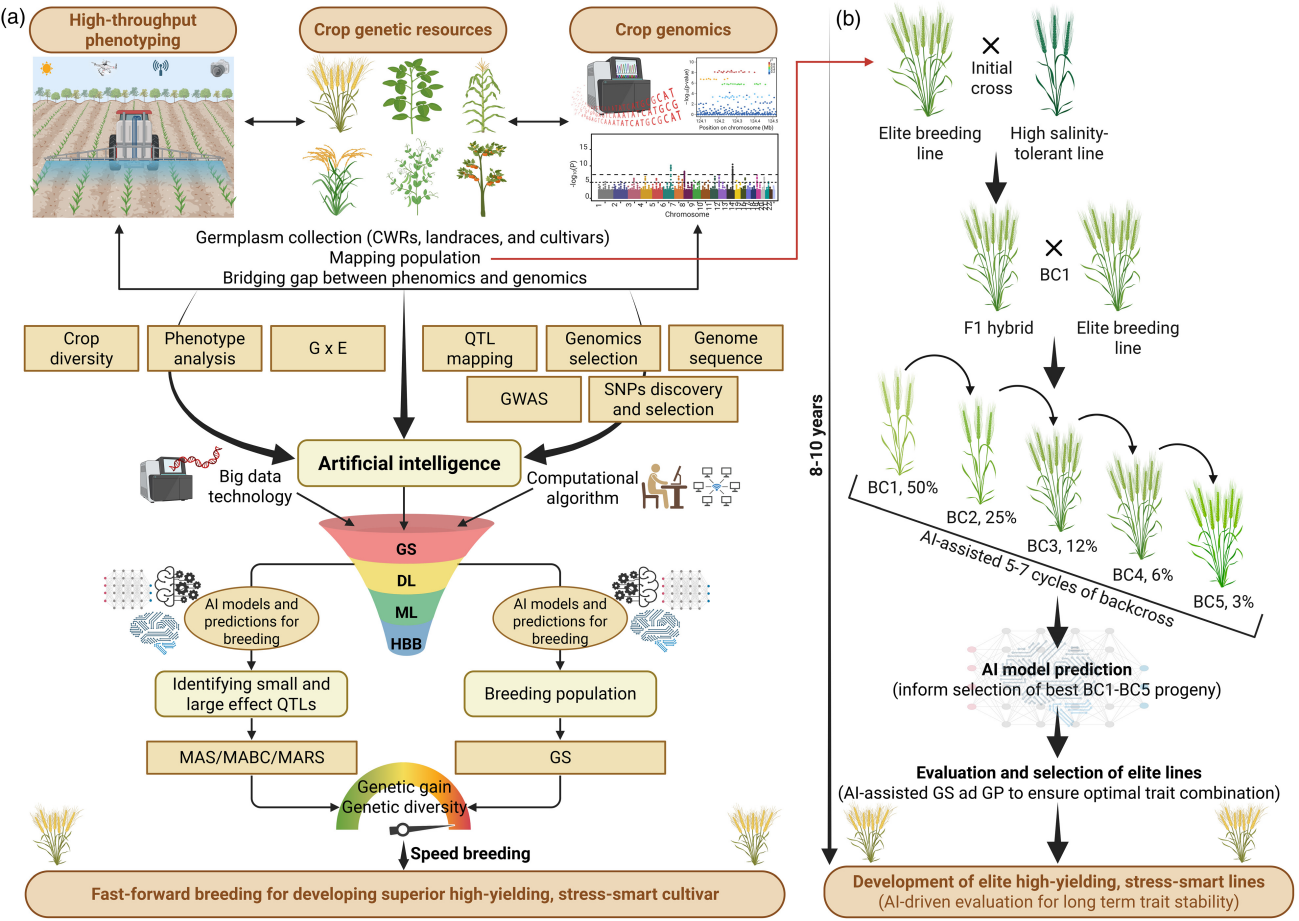
Harnessing AI and HTP for crop improvement. (a) Integrating genomics and HTP implies a transformative tool for harnessing genetic resources in crop stress breeding. This figure explains the all‐in‐one integration of genomics and HTP techniques across several breeding stages, including genome sequencing, GS, QTL mapping, GWAS, SNP discovery and selection, GxE estimation and crop diversity analysis. (b) AI‐assisted plant backcrossing. This part presents a faster breeding pipeline that integrates backcrossing cycles with AI model predictions to develop elite high‐yielding salinity‐smart breeding lines. Panel A was modified from Esposito *et al*. (2019) and Raza *et al*. (2024c) under CC BY 4.0 international licence. AI, artificial intelligence; BC, backcrossing; CRWs, crop wild relatives; DL, deep learning; GAB, genomics‐assisted breeding; GP, genomic prediction; GS, genomic selection; GWAS, genome‐wide association studies; GxE, genotype‐by‐environment estimation; HBB, haplotype‐based breeding; HTP, high‐throughput phenotyping; ML, machine learning; QTL, quantitative trait loci; SNP, single nucleotide polymorphism.

Revolutionary NGS tools for genome sequencing and HTP of crops, coupled with progress in computational tools, are transforming plant breeding (Figure 4) (Araus *et al*., 2022; Raza *et al*., 2024a; Varshney *et al*., 2021b, 2021c). These advances are assisting the discovery of genetic foundations of traits with exceptional precision. Nonetheless, current genome sequence data remain underutilized for leveraging complex multi‐gene traits due to inadequate phenotypic data (Araus *et al*., 2022; Jin *et al*., 2020). Efficient, computerized/automated and precise tools to capture and associate phenotypic data with genomic insights are necessary for crop advancement at all growth phases. Consequently, HTP has emerged as a severe bottleneck in fast‐tracking crop breeding (Song *et al*., 2021). In this context, new HTP tools and AI (particularly machine learning; ML) are playing crucial roles in biological big data mining and analysis (Esposito *et al*., 2019; Singh *et al*., 2021; Sinha *et al*., 2023; Yang *et al*., 2020; Yoosefzadeh Najafabadi *et al*., 2023). These tools deliver imperative insights for decision‐making targeted at achieving breeding objectives, exclusively in recognizing plant responses to stress such as salinity with the help of HTP (Figure 4) (Araus *et al*., 2022; Esposito *et al*., 2019; Singh *et al*., 2021; Sinha *et al*., 2023; Yang *et al*., 2020). As presented in Figure 4, high‐throughput phenotypic and genotypic data are gathered from diverse crop germplasm (including CWRs, landraces and cultivars) and breeding populations, contributing to an inclusive database enriched with valuable resources to bridge a gap between genomics and phenomics. By simulating genetic gain from several breeding strategies over manifold generations, AI permits breeders to forecast long‐term outcomes and generate informed choices on which lines to backcross. Models that examine the accumulation rate of beneficial alleles assist in designing breeding cycles more efficiently. Furthermore, AI can examine and manage genetic diversity by investigating the genetic makeup of breeding lines, find prospective inbreeding risks and confirm that the breeding program remains robust and flexible to the changing climate. AI‐driven genomic and phenomic analysis can substantially boost the efficiency and precision of backcrossing into elite breeding lines, which will lead to enhanced genetic gain and sustainability of breeding programs. Moreover, recent advancements in computational algorithms, big data technology and speed breeding further expand the proficiency and effectiveness of AI‐driven breeding approaches (e.g. MAS, GS, etc.), hence fast‐forwarding the designing of superior salinity‐smart crop varieties (Figure 4).

Literature features the power of ML and HTP (using whole‐plant‐based phenotyping systems, as discussed below) in producing valuable data for designing salinity‐smart plants. For instance, a previous study employed hyperspectral imaging and ML techniques to rank SST in wheat quantitatively (Moghimi *et al*., 2018). By classifying spectral endmembers and applying novel similarity measurement methods, the authors effectively connected spectral patterns with SST rankings, supporting the capacity of ML in non‐invasive plant phenotyping for SST (Moghimi *et al*., 2018). Feng *et al*. (2020) exploited hyperspectral imaging together with deep learning (DL) methods to examine 13 okra (*Abelmoschus esculentus* L.) genotypes against salinity conditions. Results showed that salinity altered the physiological and biochemical traits, resulting in significant shifts in the spectral data and emphasized the certainty of whole‐plant‐based phenotyping. In short, this study observed diverse responses among genotypes and authorized the functionality of HTP for evaluating physiological and biochemical traits for SST in okra (Feng *et al*., 2020). Another study investigated the temporal responses of 191 *A. thaliana* accessions to salinity stress. Employing HTP and ML algorithms, they identified key physiological traits influencing growth maintenance, enhancing our understanding of the molecular background of early plant responses to salinity stress (Awlia *et al*., 2021). The molecular mechanism behind the high SST of *Spartina alterniflora* (a halophyte) was explored by leveraging DL methods; the authors discovered 16 *SaHKTs* genes and characterized their expression patterns and ion transport properties under salinity. The potential of DL in discovering functional genes for breeding salinity‐smart crops was also demonstrated (Yang *et al*., 2023b). Recently, Vello *et al*. (2024) explored image‐based phenotyping to evaluate *Camelina sativa* seed quality non‐destructively under salinity stress. Through SeedML, an easy web tool, the authors detected key morpho‐colorimetric attributes in *C. sativa* seeds grown under high salinity conditions. This approach improves quality control, identifies stress markers and performs yield trends effectively. Overall, these findings provided new insights for stress adaptation and improved quality control measures in food production (Vello *et al*., 2024). Taken together, the above‐discussed studies feature the power of integrating HTP and ML methods for fast‐tracking the discovery and development of salinity‐smart crop plants.

Advanced AI‐based algorithms are transforming crop modelling by anticipating genetic gain and assisting accurate yield simulations under diverse environmental conditions, including salinity stress (Figure 4). By integrating QTL/GWAS and ML algorithms, scientists can discover diverse genetic variations associated with diverse traits, including SST, accelerating stress‐smart breeding programs (Esposito *et al*., 2019; Sinha *et al*., 2023; Varshney *et al*., 2017, 2021c; Yoosefzadeh Najafabadi *et al*., 2023). This collaboration between AI/ML and GAB (i.e. GS‐based prediction) holds massive potential for advancing crop efficiency and tolerance while reducing the time and costs involved in breeding efforts (Esposito *et al*., 2019; Singh *et al*., 2021; Sinha *et al*., 2023; Varshney *et al*., 2017, 2021c; Yoosefzadeh Najafabadi *et al*., 2023). Moreover, the AI‐enabled high‐throughput framework allowed real‐time digital assessment of stress‐related traits, marker identification, GS‐based prediction and enhanced genetic gain. These abilities substantially impact plant‐breed stress surveying applications (Libbrecht and Noble, 2015; Schrider and Kern, 2018).

As discussed earlier and in Table 2, GWAS/QTL have identified various important genes correlated with SST. Nevertheless, due to the complex environment of stress responses in plants, these responses have been re‐attributed to several interrelating genetic variations that are generally unnoticed in GWAS. In this context, ML algorithms can discover these genetic variants underlying SST in plants. For instance, a comprehensive analysis of 191 *A. thaliana* accessions, HTP, multivariate analysis and ML algorithms uncovered that quantum yield and maximum quantum yield extensively influence growth maintenance under salinity conditions (Awlia *et al*., 2021). Using GWAS, the authors detected 770 loci specific to SST, with two loci correlated with quantum yield and maximum quantum yield. Overall, this integrative approach combining HTP, multivariate analysis and GWAS delivers a valuable understanding of the genetic factors of SST, providing a way forward for targeted stress‐smart breeding strategies (Awlia *et al*., 2021). AI also enabled the choice of common bean genotypes with superior phenotypic stability under altered conditions (Corrêa *et al*., 2016). In short, the artificial neural networks model was used for GS‐based prediction modelling.

GS driven by ML algorithms suggests a hopeful alternative to MAS for quantitative traits by evaluating the overall genetic potential of individuals rather than focusing on specific QTLs. Recent examinations have supported the effectiveness of GS models, achieving projection correctness ranging from 0.28 to 0.62 for grain yield and stress tolerance in elite wheat breeding lines (Poland *et al*., 2012; Rutkoski *et al*., 2013). Nonetheless, GS faces challenges related to prediction accuracy flexibility. Addressing this limitation requires enhanced data sharing, integration efforts and the development of novel prediction models to ensure accuracy. For instance, Bernardo and Yu (2007) showed that utilizing the complete set of available markers for genotyping allows for a more accurate projection of breeding values than subsets associated with specific QTLs in maize. Accordingly, Heffner *et al*. (2011) analytically validated this finding by evaluating the predictive performance of phenotypic selection, MAS and GS across 13 phenotypic traits in wheat breeding lines using whole‐plant‐based phenotyping. In alfalfa, marker analysis and GS models identified SNP markers associated with SST and anticipated breeding values using ML methods, resulting in the highest yield prediction accuracy (0.793) (Medina *et al*., 2020). These outcomes improve our knowledge for enhancing SST in alfalfa and fast‐tracking the development of stress‐smart cultivars with the help of GAB and ML methods (Medina *et al*., 2020). To fully harness the power of these integrated approaches, interdisciplinary collaborations, such as computational biology, agronomy and plant physiology, are essential. We argue that these collaborations could enable the development of unique phenotyping pipelines, improve data explanation and enhance the practical application of AI‐driven breeding strategies in actual agricultural backgrounds.

**Figure 5:**
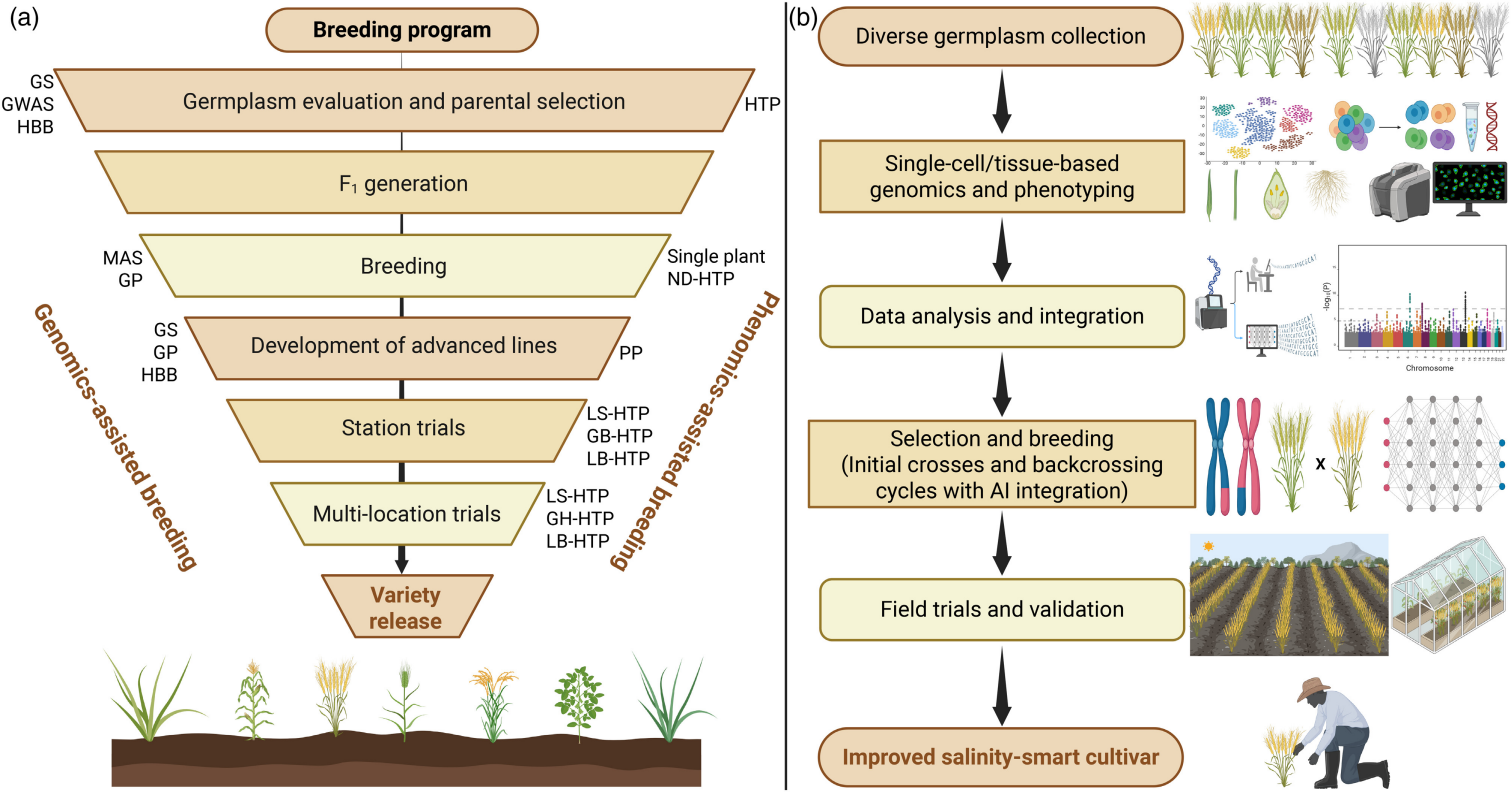
Current and proposed breeding pipelines. (a) A stepwise presentation of a current breeding program to release improved crop varieties by integrating genomics‐ and phenomics‐assisted breeding. This pipeline concludes with a variety release after the comprehensive trials, which can be readily available to the farmers. (b) The new proposed breeding pipeline profits from fast‐forward testing technologies, for example, single‐cell/tissue‐based comprehensive genomics and phenotyping to swiftly and non‐destructively evaluate SST in diverse germplasm (including elite lines, high salinity‐tolerant lines, CWRs, landraces, etc.) in actuality. In short, both breeding pipelines help design the development of high‐performing, high‐yielding and salinity‐smart future crop varieties, which, in turn, fast‐track the breeding program for enhanced productivity. CWRs, crop wild relatives; GP, genomic prediction; GS, genomic selection; GWAS, genome‐wide association studies; GH‐HTP, greenhouse‐based HTP; HTP, high‐throughput phenotyping; LB‐HTP, laboratory‐based HTP; LS‐HTP, large‐scale HTP; MAS, marker‐assisted selection; ND‐HTP, non‐destructive HTP; PP, phenomic prediction; SST, salinity stress tolerance.

Advanced AI algorithms modernize crop modelling, assisting precise yield predictions under salinity stress. Leveraging ML algorithms and GAB to discover hidden genetic variants (mainly GS‐based prediction) underlying SST and combining HTP present valuable ways forward in stress tolerance genetics. Moreover, integrating HTP platforms with genomic heritability and ecological collaborations is necessary, and AI bridges the gap between genome and phenome. Moreover, the integration of AI model predictions with traditional backcrossing cycles can notably fast‐track the development of elite high‐yielding and salinity‐smart breeding lines (Figure 4b). As detailed in Figure 4b, this can be practically implemented through a breeding pipeline that begins with initial crosses between an elite breeding line (high‐yielding) and high salinity‐tolerant lines to combine desirable traits from both parents. The resulting progeny are then repetitively backcrossed (typically 5–7 cycles) with the elite parent to maintain yield traits while progressively enhancing SST. At each cycle, AI models predict the best candidates for further breeding by examining genetic data and environmental factors to spot the most promising lines. Field trials should be performed to validate performance under saline conditions, and AI‐supported GS and GP approaches ensure long‐term trait stability and adaptability in natural environments. Likewise, integrating HTP (or phenomics‐assisted breeding ‘PAB’) and GAB methods for widespread phenotype evaluation suggests new avenues for fast‐tracking stress‐smart breeding and variety release (Figure 5a). As shown in Figure 5a, at the germplasm evaluation and parental selection stage, GAB leverages GS, GWAS and HBB to discover and rapidly choose the best parental lines (i.e. high‐yielding and salinity‐smart) based on their genetic potential, thus fast‐tracking the selection process. At the same time, PAB employs HTP technologies to evaluate phenotypic traits within large populations. During breeding, GAB uses MAS and GP to improve selection based on precise genetic markers and traits. PAB leverages ND‐HTP techniques to observe the plant's health and performance simultaneously without breaking them. During the development of advanced lines, GAB integrates GS, GP and HBB to discover superior lines and calculate future performance. Mainly, HBB can be key in discovering and combining superior haplotypes into breeding lines and contribute to improved stress tolerance and other anticipated traits. In comparison, PAB applies PP estimation to assess and compare phenotypic characteristics. This stage also embraces iterative backcrossing into elite breeding lines to combine salinity tolerance and high yield potential. For station and multi‐location trials, PAB employs LS‐ (e.g. UAV‐ and rover‐based), GH‐ and LB‐HTP systems to generate inclusive data on how the varieties perform under diverse environmental conditions, which enables the selection of high‐performing lines (Figure 5a). In short, the integration of GAB, AI and HTP techniques is considered a fast‐forward tool for designing stress‐smart crop plants and future breeding methods.

### Drilling down from whole‐plant to single‐cell/tissue: Overcoming phenotyping bottlenecks

A major bottleneck in breeding for SST is not genotyping but accurate and appropriate phenotyping. Traditional phenotyping methods mainly count on whole‐plant evaluations, which fail to portray the detailed cell‐ and tissue‐specific responses necessary for understanding and advancing SST (Araus *et al*., 2022; Jin *et al*., 2020; Song *et al*., 2021). The advantage of whole‐plant‐based phenotyping is its high throughput and relatively low cost. This comes at the expense of the accuracy and predictive value, as this approach does not account for the cell‐ and tissue‐specific operation of specific traits/genes. These limitations are intensified by the lack of funding for advanced phenotyping techniques and the influence of companies advocating more swiftly available but less precise phenotyping tools. Several experiments have underlined these limitations. For instance, while hyperspectral imaging and ML techniques have been used to rank SST in wheat (Moghimi *et al*., 2018), the reliance on whole‐plant measurements can ignore significant cellular stress responses. Likewise, studies on okra (Feng *et al*., 2020) and *Arabidopsis* (Awlia *et al*., 2021) using whole‐plant phenotyping have delivered significant insights but failed to secure complete cellular mechanisms. By using CoroNa Green fluorescence imaging, Wu *et al*. (2015) showed that cytosolic Na^+^ levels in meristematic root cells of salt‐tolerant wheat varieties were fourfold higher than in sensitive genotypes, as this tissue harbours putative salt sensors (Wu *et al*., 2018). Thus, if one relies on whole‐root Na^+^ analysis, these varieties with superior sensing mechanisms will be missed. The use of DL to evaluate SST in *Spartina alterniflora* (Yang *et al*., 2023b) and image‐based phenotyping in *Camelina sativa* (Vello *et al*., 2024) also highlights the need for more precise, cell‐specific investigations to fully harness the genetic and physiological responses to manage SST. Hence, transitioning towards sc‐based phenotyping is necessary for discovering the detailed cellular mechanisms underlying SST.

In a similar context, sc‐genomics also offers a more detailed and precise comprehension of stress responses by focusing on single cells and tissues. This method can discover the specific cellular mechanisms and pathways that participate in SST, thereby delivering more targeted and efficient breeding approaches. However, it should be noted that trusting a single trait as an alternative for tolerance incorporates significant risk and is questionable to be trustworthy. In this context, a multiparametric screening method can be a promising hope, though at the cost of high throughput. Nonetheless, a transformative key remains in advancing and implementing cell‐based phenotyping platforms, which can play a significant role in fast‐tracking future breeding programs (Kotula *et al*., 2024). For instance, investigating the whole shoot or leaf for Na^+^ content fails to account for its intracellular distribution (Panta *et al*., 2014). Cell‐based screening methods, for example, fluorescent CoroNa Green dye (Cuin *et al*., 2011) or high‐resolution cryo‐scanning electron microscopy X‐ray microanalysis (Oi *et al*., 2022), distinguish between vacuolar and cytosolic/chloroplastic Na^+^ distribution, establishing cultivars as truly tolerant if they accumulate higher quantities of leaf Na^+^ (measured by ICP spectroscopy or flame photometer) while proficiently sequestering it in vacuoles (measured by CoroNa Green florescent dye or high‐resolution cryo‐scanning electron microscopy X‐ray microanalysis). In another study, Wu *et al*. (2015) demonstrated the effectiveness of using confocal laser scanning microscopy with CoroNa Green fluorescent dye imaging to screen wheat cultivars for their capability to sequester Na^+^ in root vacuoles. They also examined six wheat varieties (three tolerant and three sensitive) to understand tissue‐specific Na^+^ sequestration and its influence on overall SST and determined the capacity of CoroNa Green fluorescent dye for picking donors for future breeding programs (Wu *et al*., 2015). These findings highlight the potential of tissue‐specific phenotyping in detecting key traits and mechanisms for SST, which can substantially enhance breeding programs. The ability of plant roots to prevent NaCl‐induced K^+^ loss from the root is vital for SST. Therefore, assessment of NaCl‐induced K^+^ efflux from specific types of root cells may be exploited as an extremely efficient screening tool, acquiring significant genetic variability in SST, for example, in barley (Chen *et al*., 2007).

Earlier, Gill *et al*. (2017) assessed the plasma membrane potential of epidermal root cells via cell‐based phenotyping using the microelectrode MIFE method in barley. They identified a major QTL on Chr2H for the membrane potential in the root cells, demonstrating the power of the sc‐based phenotyping, contributing to salinity and waterlogging tolerance in barley and delivering new prospects for fine mapping in MAB (Gill *et al*., 2017). In another study, cell‐based phenotyping was evaluated using two root cell viability markers (e.g. viability assay and root growth assay) as substitutes for the technically problematic MIFE method using barley (Wang *et al*., 2019a). Image‐based phenotyping of single root cells exhibited that the viability of salinity‐tolerant barley was less influenced by long‐term ROS exposure than was salinity‐sensitive barley varieties. A substantial negative association was found between relative root length reduction and overall SST. These discoveries recommend that oxidative and SST in barley can be successfully screened using either fluorescein diacetate and propidium iodide staining or low‐cost root length measurements, suggesting suitable methods for breeders to aim at root‐based genes regulating ion homeostasis. Furthermore, crosses between tolerant and sensitive lines could assist in discovering QTL aiding ROS tolerance in root cells during salinity stress (Wang *et al*., 2019a).

Advocating for higher investment in advanced phenotyping methods, mainly emphasizing the need for cell‐ and tissue‐specific analysis, permits us to push for a more advanced approach in breeding salinity‐smart crops, transitioning from whole‐plant to sc‐based phenotyping (Figure 5b). This strategic shift is vital for precisely selecting and breeding genotypes with superior SST, which is critical for confirming sustainable agricultural productivity. Despite their higher cost, methods like confocal fluorescent microscopy offer vital insights into ion compartmentalization and other important cellular processes related to SST (Lee *et al*., 2016; Wu *et al*., 2015). Combining these advanced phenotyping methods with genomics tools can enhance our knowledge of the genetic and physiological basics of SST, which will assist in developing more informed breeding strategies. Moreover, these new tools can improve efforts to improve high SST in plants by implementing modified new breeding strategies. For instance, developed genetic tools (e.g. GWAS, map‐based cloning, etc.) facilitate fast screening of huge populations for new variants by linking phenotypes with genotypes. Subsequently, certain lines undergo backcrossing cycles supported by AI model predictions (see Figure 4 for insights on AI's role in breeding elite lines) that introduce high SST alleles into high‐yielding lines followed by multi‐site field trials. This pipeline targets high SST much earlier in the breeding procedure by leveraging the highest genetic potential of germplasms (Figure 5b). In short, early integration of sc‐cell/tissue‐based genomics and phenotyping to select ideal yield‐salinity trade‐offs, genetic screening of wild germplasm, and evolving new populations to recognize genetic variations for breeding elite high‐yielding, salinity‐smart cultivars (Figure 5b).

## Concluding remarks and way forwards

This review explores the recent advances in fast‐forward and modern GAB tools that offer ways forward to design salinity‐smart future crop varieties (Figures 2, 3, 4, 5). These innovative breeding designs, coupled with genomic technologies, are a transformative era of stress‐smart breeding programs. Notably, recent innovations in genomics research have significantly enhanced precision and efficiency, supporting geneticists, biologists and breeders with diverse tools and technologies to fast‐track the breeding landscape and improve trait selection. Furthermore, the success of GAB is the precise phenotyping of salinity‐related traits across diverse breeding materials. It is also decisive to highlight the need for advancements in phenotyping methods, mainly towards single‐cell or tissue‐specific analysis, to overcome the limitations of whole‐plant assessments. Dissecting phenotypes into components enhances heritability and promotes a deeper understanding of the biological structures underlying stress responses.

Moreover, the availability of reference genome assemblies for naturally salinity‐tolerant plants and CWRs fast‐track gene discovery and trait manipulation methods. Therefore, we argue that stress‐smart cultivars should be acted and delivered ‘from gene banks to farmer's field’ by harnessing the full power of close/distant CWRs. In the future, more efforts will be required to fully harness the potential of these natural resources and transfer their traits into today's cultivated crops to make them thrive in response to high salinity conditions (Figure 3). Additionally, GAB‐based speed breeding can be exploited to advance rapidly improved generations in a short period.

Sc‐genomics/sc‐RNA‐seq and epigenomics are advanced tools for investigating cellular and epigenetic mechanisms underlying stress responses with exceptional resolution. Single‐cell methods can discover stress‐responsive pathways and cell‐specific regulatory networks that are frequently obscured in bulk tissue analysis. On the other hand, epigenomics delivers insights into heritable stress adaptations, such as DNA methylation and histone modifications, which play key roles in regulating gene expression under salinity stress. Moving forward, more research is required to fully harness the power of both methods in cultivated food crops and CWRs that can help design future crops with improved salinity tolerance.

With the arrival of high‐throughput marker development, genotyping procedures, and precision HTP, GAB stands self‐confident in harnessing the genetic landscape swiftly and effectively. Nevertheless, the genetic and genomic approaches emphasize significant enhancements in breeding activities, and their integration with conventional methodologies remains imperative. Looking ahead, the synergistic utilization of MAB, GS and other GAB tools promises to accelerate the stress‐smart breeding efforts at the genomic level. Also, targeted transgenomics, gene editing or introgression breeding can aid in recovering domesticated features while preserving valuable remarkable traits via *de novo* domestication.

MAB/MAS has been able to deliver insights into SST across diverse plant species (Table 2); yet, the scarcity of understanding concerning specific gene action in *planta* persists as a foremost challenge in breeding events that highlight the stipulation to develop cell‐based phenotyping methods. Nevertheless, boosting genetic gains permits speedy population improvement. In this context, pan‐genomics, GS, HBB, HTP (mainly single organ/tissue‐based phenotyping) and AI tools are progressively being used to support genomic predictions and boost genetic gains for fast‐tracking the breeding of salinity‐smart plants (Figure 4). Moreover, integrating these superior phenotyping methods with genomics tools can boost our perception of SST mechanisms, opening the way for more targeted breeding schemes (Figure 5b).

Through precise links between genotypes and phenotypes, AI algorithms fast‐track the gene discovery linked to SST, advancing breeding programs and paving the way forward for designing future crop varieties. In the coming years, the integration of GAB with AI and HTP is estimated to play a dynamic role in breeding more stress‐smart crop cultivars with elevated nutritional rates in an economical and well‐timed manner. Thus, more efforts are required to guarantee that these fast‐forward tools are reachable and reasonable to modest farmers, particularly in evolving countries where modern crops are needed to meet food and energy demands.

To enable these advancements, support breeders and fast‐track breeding efforts, many exciting online databases, mainly integrating AI/ML, GS, HTP, etc., have been established including Smart Breeding Platform (https://sbp.ibreed.cn): providing insights into high‐throughput population genetics, phenomics and GS (Li *et al*., 2024b); BreedingAIDB (http://ibi.zju.edu.cn/BreedingAIDB): integrating crop genome‐to‐phenotype paired data with ML tools appropriate for breeding (Shen *et al*., 2024); CropGS‐Hub (https://iagr.genomics.cn/CropGS): genotype–phenotype resources for genomic estimate in major crops for fast‐tracking genome‐designed breeding (Chen *et al*., 2024); CLIMtools (https://gramene.org/CLIMtools/index.html): resource for pan‐genome estimate of climate‐related genetic variants in crops (Ferrero‐Serrano *et al*., 2024); Crop‐GPA (https://crop‐gpa.aielab.net): platform for crop gene‐phenotype association information (Gao *et al*., 2024), and many other crop‐specific databases. These freely available resources can guide and facilitate breeders in fast‐tracking stress‐smart breeding and designing future crops in both resource‐limited and well‐funded regions/institutions to enhance their practicality. Nevertheless, ongoing improvements in bioinformatics databases remain imperative for finding stress‐associated candidates and designing nutritionally rich and stress‐smart crops personalized for future generations. Despite comprehensive advances in GAB and other tools, several essential questions and bottlenecks still entail continued devotion and rigorous efforts from plant breeders to design stress‐smart future cultivars (*see outstanding questions*).

### Outstanding questions

1. Could salinity‐tolerant plants (such as halophytes, grasses, etc.) and CWRs offer sustainable solutions for food production in saline‐affected lands?
2. How can we leverage the vast genetic diversity of CWRs to design novel breeding plans for improving salinity tolerance in modern cultivated crops?
3. How can we systematically discover the complex crosstalk between salinity and other co‐occurring stresses (combined abiotic stresses) at the molecular, physiological and metabolic levels to develop breeding and biotechnological strategies for stress‐smart crop improvement? Can panomics be the solution?
4. What are the vital genomic regions and alleles associated with salinity tolerance in naturally occurring salinity‐tolerant plants and how can this knowledge be successfully translated into breeding programs for major food crops? Can pan‐genomics be the solution?
5. Considering the complications of salinity tolerance, what approaches can be commissioned to resourcefully handle the integration of multiple genes in breeding programs, and how can modern genomics and bioinformatics tools help in this process?
6. Given the context‐specific nature of gene expression for salinity tolerance, how can breeders tailor gene combinations to address variable salinity levels and environmental conditions, and what role does the “horses for courses” approach play in this process?
7. What are the most effective approaches for discovering and introgressing salinity tolerance traits from wild plant species into modern crop varieties while guaranteeing the preservation of anticipated agronomic traits during this process?
8. What novel genomic landscape and regulatory elements can pan‐genomics discover to enhance our capability to engineer salinity‐smart crop plants?
9. What insights can single‐cell genomics deliver into molecular mechanisms triggering salinity responses at the cellular level in different plant tissues?
10. How can pan‐genomics and single‐cell genomics facilitate the discovery of genetic markers and regulatory networks accompanying salinity tolerance traits, enabling MAS and genomic predictions in future breeding programs?
11. How can we optimize GS methodologies to effectively harness the vast genetic diversity present in CWRs and new germplasm collections, permitting the rapid development of stress‐smart crop varieties?
12. In haplotype‐based breeding, how can we integrate novel genetic relations documented by relocated haplotypes with the beneficiary genetic background, ensuring the development of designer cultivars with improved salinity tolerance while preserving desirable agronomic traits?
13. What novel and advanced genomic tools can be acquired to overcome the constraints of conventional breeding approaches and fast‐track the design of salinity‐smart crop varieties?
14. How can advanced phenotyping tools (whole‐plant or tissue/cell‐based assessment) and AI be harnessed to fast‐track the effectiveness and accuracy of stress‐smart breeding programs?
15. Can crops designed using data from HTP and AI achieve the same level of success as those designed exclusively through GAB? Can AI and HTP further transform and accelerate the GAB process, ultimately leading to the rapid advancement of stress‐smart crop varieties?

## Author contributions

AR, ZH and RKV conceived the idea and designed the project. AR prepared the original draft and designed the figures and tables with inputs from all authors. QUZ helped with the literature search and discussion. SS, MT, ZH, RM and RKV critically reviewed, provided insightful suggestions and edited the manuscript. ZH and RKV supervised the work. All authors have read and approved the final version of the manuscript.

## Funding

ZH's work was supported by the National Natural Science Foundation of China (grant no. 32273118), Guangxi Major Program for Science and Technology (grant no. GuikeAA24263042), Shenzhen Special Fund for Sustainable Development (grant no. KCXFZ20211020164013021), Guangdong Key R & D Project (grant no. 2022B1111070005), The Engineering Research Center Support Program from the Development and Reform Commission of Shenzhen Municipality (XMHT20220104019), the Shenzhen University 2035 Program for Excellent Research (2022B010) and Guangdong Provincial Key Laboratory of Functional Substances in Medicinal Edible Resources and Healthcare Products (2021B1212040015). RKV's work was supported by the Food Futures Institute (FFI) of Murdoch University as well as the Grains Research & Development Corporation for supporting research projects on the development of genomics and pre‐breeding research in wheat (grant nos. UMU2404‐003RTX and WSU2303‐001RTX) and legumes (grant nos. UMU2403‐009RTX and UMU2303‐003RTX).

## Conflict of interest

The authors declare no conflict of interest.

## Supporting information


**Table S1** Salinity stress reduces the growth and yield of various plant species.

**Table S2** An overview of infrastructure, equipment and expertise needed for different levels of genomic‐assisted breeding applications for crop improvement programs.

## Data Availability

Data sharing is not applicable to this article as no data sets were generated or analysed during the current study.
