# Supplementary material for: Genomics‐assisted breeding for designing salinity‐smart future crops
Source: Plant Biotechnol J. 2025 May 20;23(8):3119–51. doi: 10.1111/pbi.70104 (PMC12310839; doi:10.1111/pbi.70104)
Supplement: Supplementary file 1 — Table S1 Salinity stress reduces the growth and yield of various plant species. [file PBI-23-3119-s001.docx]

**Genomics-assisted breeding for designing salinity-smart future crops**

Ali Raza^1,2^, Qamar U Zaman^3^, Sergey Shabala^4,5^, Mark Tester^6^, Rana Munns^7^, Zhangli Hu^1,2,8*^, Rajeev K. Varshney^9*^

^1^Guangdong Key Laboratory of Plant Epigenetics, College of Life Sciences and Oceanography, Shenzhen University, Shenzhen, China

^2^Shenzhen Engineering Laboratory for Marine Algal Biotechnology, Guangdong Technology Research Center for Marine Algal Biotechnology, Longhua Innovation Institute for Biotechnology, College of Life Sciences and Oceanography, Shenzhen University, Shenzhen, China

^3^School of Breeding and Multiplication, Hainan Yazhou Bay Seed Laboratory, Hainan University, Sanya, China

^4^School of Biological Sciences, The University of Western Australia, Perth, WA, Australia

^5^International Research Centre for Environmental Membrane Biology, Foshan University, Foshan, China

^6^Center of Excellence for Sustainable Food Security and Division of Biological and Environmental Sciences and Engineering, King Abdullah University of Science and Technology (KAUST), Thuwal, Saudi Arabia

^7^Centre of Excellence in Plant Energy Biology, School of Molecular Sciences, The University of Western Australia, Perth, WA, Australia

^8^Guangdong Provincial Key Laboratory of Functional Substances in Medicinal Resources and Healthcare Products, School of Life Sciences and Food Engineering, Hanshan Normal University, Chaozhou, China

^9^WA State Agricultural Biotechnology Centre, Centre for Crop and Food Innovation, Food Futures Institute, Murdoch University, Murdoch, WA, Australia

**^*^Corresponding author**: [huzl@szu.edu.cn](mailto:huzl@szu.edu.cn) / [rajeev.varshney@murdoch.edu.au](mailto:rajeev.varshney@murdoch.edu.au)

**Table S1.** Salinity stress reduces the growth and yield of various plant species.

| **Plant specie** | **Stress condition** | **Experimental condition** | **Key effects in terms of reduction/decrease** | **Reference** |
| --- | --- | --- | --- | --- |
| **Growth-related traits** | | | | |
| Wheat (*Triticum aestivum* L.) | 160 mM NaCl; 21 d | Greenhouse | Roots and shoots dry weights | Pour-Aboughadareh *et al.* (2021a) |
| Wheat (*T. aestivum* L.) | 15 dS m^−1^ | Pot | Plant height, spike length, and number of spikelets spike^−1^ | Saddiq *et al.* (2020) |
| Wheat (*T. aestivum* L.) | EC of 7.17 dS m^−1^; 120 | Pot | Shoots length, roots and shoots fresh and dry weights, and spike length | Rizwan *et al.* (2023) |
| Wheat (*T. aestivum* L.) | 1.10 g L^−1^ NaCl, and 0.40 g L^−1^ Na_2_SO_4_; tillering to maturity | Field | Plant height, spike length, and number of adventitious roots, and nodal roots | Aniskina *et al.* (2023) |
| Barley (*Hordeum vulgare* L.) | 200 mM NaCl; 30 d | Greenhouse | Roots and shoots fresh/dry weights | Pour-Aboughadareh *et al.* (2021b) |
| Rice (*Oryza sativa* L.) | <2, 3-5, and 8 dS m^−1^; tillering to maturity | Field | Plant height, panicle length, and total dry weight | Santanoo *et al.* (2023) |
| Rice (*O. sativa* L.) | EC 0.55, 3.40, 6.77, and 8.00 mS cm^–1^; till reproductive stage | Greenhouse | Plant height, leaf area, and leaf fresh and dry weights | Shukry *et al.* (2023) |
| Rice (*O. sativa* L.) | 45 and 75 mM; 3 weeks | Field | Plant height, shoots fresh and dry weights, and productive tillers | Ghadirnezhad Shiade *et al.* (2023) |
| Pearl millet (*Pennisetum glaucum* L.) | EC 3, 6, and 9 dS m^–1^; till harvesting | Pot house | Plant height | Kumar *et al.* (2023) |
| Pea (*Pisum sativum* L.) | 50 and 100 mM NaCl; 60 and 75 d | Pot | Plant height | Al-Shammari *et al.* (2023) |
| Tomato (*Solanum lycopersicum* L.) | 0.5 and 30 mM NaCl | Greenhouse | Leaf dry matter content | Ntanasi *et al.* (2023) |
| Indian mustard (*Brassica juncea* L.) | 50, 100, and 150 mM NaCl; 30 d | Pot/Natural environment | Roots and shoots lengths and fresh and dry weights | Verma *et al.* (2023) |
| Sunflower (*Helianthus annuus* L.) | 100 mM NaCl; till reproductive stage | Pot | Shoots/Roots fresh and dry weight, shoot and root lengths, leaf area, and first internode length | Lalarukh *et al.* (2023) |
| Quinoa (*Chenopodium quinoa* L.) | 8, 16, and 24 dS m^−1^; till maturity | Pot house | Plant height and roots and shoots dry weights, and length of panicle | Prajapat *et al.* (2024) |
| Quinoa (*C. quinoa* L.) | ECe 15–20 dS m^−1^ | Pot and field | Plant height, and panicle length | Ejaz *et al.* (2024) |
| **Yield and associated traits** | | | | |
| Wheat (*T. aestivum* L.) | 15 dS m^−1^ | Pot | Fertile tillers, number of grains spike^−1^, 100-grain weight, grain yield, and biomass production | Saddiq *et al.* (2020) |
| Wheat (*T. aestivum* L.) | 160 mM NaCl; 21 d | Greenhouse | Grain yield | Pour-Aboughadareh *et al.* (2021a) |
| Wheat (*T. aestivum* L.) | EC of 7.17 dS m^−1^; 120 | Pot | Grain weight | Rizwan *et al.* (2023) |
| Wheat (*T. aestivum* L.) | 0.4, 4.0, 8.0, 12 dSm^−1^ diluted seawater | Pot | Grain yield pot^−1^ | Elfanah *et al.* (2023) |
| Rice (*O. sativa* L.) | <2, 3-5, and 8 dS m^−1^; tillering to maturity | Field | Number of unfilled grains panicle^−1^, weight of 100 filled grains, grain number, biomass at maturity, and filled grain weight plant^−1^ | Santanoo *et al.* (2023) |
| Rice (*O. sativa* L.) | 1.0 and 2.0 g kg^−1^ artificial sea salt; till maturity | Field | Grain yield, number of spikelets panicle^−1^, filled grains, and 1000-grain weight | Li *et al.* (2023) |
| Maize (*Z. mays* L.) | 60 and 120 mM NaCl; till maturity | Greenhouse | Number of grains cob^−1^, grains weight cob^−1^, 100-grains weight, and grain yield plant^−1^ | Seleiman *et al.* (2023) |
| Maize (*Z. mays* L.) | 25, 50, 75, and 100 mM NaCl; till harvest maturity | Field | Number of seeds, arrangement of seeds cob^−1^, and cob size plant^−1^ | Baghel *et al.* (2019) |
| Pearl millet (*Pennisetum glaucum* L.) | EC 3, 6, and 9 dS m^–1^; till harvesting | Pot house | Above ground biomass | Kumar *et al.* (2023) |
| Tomato (*S. lycopersicum* L.) | 0.5 and 30 mM NaCl | Greenhouse | Fruit weight, number of fruits plant^−1^, and marketable yield | Ntanasi *et al.* (2023) |
| Cotton (*Gossypium hirsutum* L.) | 0.05 and 0.35% saline-alkaline soil | Pot | Boll biomass, number and weight, and fiber and cottonseed yield | Sun *et al.* (2024) |
| Quinoa (*C. quinoa* L.) | 8, 16, and 24 dS m^−1^; till maturity | Pot house | Number of branches plant^−1^, number of panicles, 1000-grain weight, and grain yield plant^−1^ | Prajapat *et al.* (2024) |
| Quinoa (*C. quinoa* L.) | ECe 15–20 dS m^−1^ | Pot and field | 1000 seed weight, seed yield, and biomass yield | Ejaz *et al.* (2024) |

**Abbreviations:** days (d); deciSiemen per metre (dS m^−1^); electrical conductivity (ECe); millimolar (mM); sodium chloride (NaCl); sodium sulfate (Na_2_SO_4_).

**References**

Al-Shammari, W.B., Altamimi, H.R. and Abdelaal, K. (2023) Improvement in physiobiochemical and yield characteristics of pea plants with nano silica and melatonin under salinity stress conditions. *Horticulturae.* **9,** 711.

Aniskina, T.S., Baranova, E.N., Lebedev, S.V., Reger, N.S., Besaliev, I.N., Panfilov, A.A., Kryuchkova, V.A. and Gulevich, A.A. (2023) Unexpected effects of sulfate and sodium chloride application on yield qualitative characteristics and symmetry indicators of hard and soft wheat kernels. *Plants.* **12,** 980.

Baghel, L., Kataria, S. and Jain, M. (2019) Mitigation of adverse effects of salt stress on germination, growth, photosynthetic efficiency and yield in maize (*Zea mays* L.) through magnetopriming. *Acta Agrobotanica.* **72**.

Ejaz, M., Bakhtavar, M.A., Iqbal, S., Khan, M.A., Jabeen, R., Jabeen, N. and Raza, A. (2024) Soil Application of Potassium Maintains Growth, Water Relations, Yield and Seed Quality of Quinoa in Salt Affected Soils. *Journal of Crop Health.* **76,** 287-295.

Elfanah, A.M., Darwish, M.A., Selim, A.I., Shabana, M.M., Elmoselhy, O.M., Khedr, R.A., Ali, A.M. and Abdelhamid, M.T. (2023) Spectral reflectance indices’ performance to identify seawater salinity tolerance in bread wheat genotypes using genotype by yield* trait biplot approach. *Agronomy.* **13,** 353.

Ghadirnezhad Shiade, S.R., Pirdashti, H., Esmaeili, M.A. and Nematzade, G.A. (2023) Biochemical and physiological characteristics of mutant genotypes in rice (*Oryza sativa* L.) contributing to salinity tolerance indices. *Gesunde Pflanzen.* **75,** 303-315.

Kumar, A., Sheoran, P., Mann, A., Yadav, D., Kumar, A., Devi, S., Kumar, N., Dhansu, P. and Sharma, D.K. (2023) Deciphering trait associated morpho-physiological responses in pearlmillet hybrids and inbred lines under salt stress. *Frontiers in Plant Science.* **14,** 1121805.

Lalarukh, I., Zahra, N., Shahzadi, A., Hafeez, M.B., Shaheen, S., Kausar, A. and Raza, A. (2023) Role of aminolevulinic acid in mediating salinity stress tolerance in sunflower (*Helianthus annuus* L.). *Journal of Soil Science and Plant Nutrition.* **23,** 5345-5359.

Li, Z., Zhou, T., Zhu, K., Wang, W., Zhang, W., Zhang, H., Liu, L., Zhang, Z., Wang, Z. and Wang, B. (2023) Effects of salt stress on grain yield and quality parameters in rice cultivars with differing salt tolerance. *Plants.* **12,** 3243.

Ntanasi, T., Karavidas, I., Zioviris, G., Ziogas, I., Karaolani, M., Fortis, D., Conesa, M.À., Schubert, A., Savvas, D. and Ntatsi, G. (2023) Assessment of Growth, Yield, and Nutrient Uptake of Mediterranean Tomato Landraces in Response to Salinity Stress. *Plants.* **12,** 3551.

Pour-Aboughadareh, A., Mehrvar, M.R., Sanjani, S., Amini, A., Nikkhah-Chamanabad, H. and Asadi, A. (2021a) Effects of salinity stress on seedling biomass, physiochemical properties, and grain yield in different breeding wheat genotypes. *Acta Physiologiae Plantarum.* **43,** 98.

Pour-Aboughadareh, A., Sanjani, S., Nikkhah-Chamanabad, H., Mehrvar, M.R., Asadi, A. and Amini, A. (2021b) Identification of salt-tolerant barley genotypes using multiple-traits index and yield performance at the early growth and maturity stages. *Bulletin of the National Research Centre.* **45,** 117.

Prajapat, K., Sanwal, S.K. and Sharma, P.C. (2024) Screening of quinoa (*Chenopodium quinoa* Willd.) germplasms under high-SAR saline water on the basis of growth, yield, and multivariate analysis. *Journal of Biosciences.* **49,** 23.

Rizwan, M., Ahmad, S. and Ali, S. (2023) Combined effect of Zinc lysine and biochar on growth and physiology of wheat (*Triticum aestivum* L.) to alleviate salinity stress. *Frontiers in Plant Science.* **13,** 1017282.

Saddiq, M.S., Afzal, I., Basra, S.M., Iqbal, S. and Ashraf, M. (2020) Sodium exclusion affects seed yield and physiological traits of wheat genotypes grown under salt stress. *Journal of Soil Science and Plant Nutrition.* **20,** 1442-1456.

Santanoo, S., Lontom, W., Dongsansuk, A., Vongcharoen, K. and Theerakulpisut, P. (2023) Photosynthesis Performance at Different Growth Stages, Growth, and Yield of Rice in Saline Fields. *Plants.* **12,** 1903.

Seleiman, M.F., Ahmad, A., Alhammad, B.A. and Tola, E. (2023) Exogenous application of zinc oxide nanoparticles improved antioxidants, photosynthetic, and yield traits in salt-stressed maize. *Agronomy.* **13,** 2645.

Shukry, W.M., Abu-Ria, M.E., Abo-Hamed, S.A., Anis, G.B. and Ibraheem, F. (2023) The efficiency of humic acid for improving salinity tolerance in salt sensitive rice (*Oryza sativa*): growth responses and physiological mechanisms. *Gesunde Pflanzen.* **75,** 2639-2653.

Sun, L., Wang, Z., Xiong, C., Gu, J., Zheng, Y., Ju, F., Wang, S., Hu, W., Zhao, W. and Zhou, Z. (2024) Improving the soil K+/Na+ ratio under moderate salt stress synergistically increases the yield and quality of cotton fiber and cottonseed. *Industrial Crops and Products.* **213,** 118441.

Verma, T., Bhardwaj, S., Raza, A., Djalovic, I., Prasad, P.V. and Kapoor, D. (2023) Mitigation of salt stress in Indian mustard (*Brassica juncea* L.) by the application of triacontanol and hydrogen sulfide. *Plant Signaling & Behavior.* **18,** 2189371.
