# Supplementary material for: Genomics‐assisted breeding for designing salinity‐smart future crops
Source: Plant Biotechnol J. 2025 May 20;23(8):3119–51. doi: 10.1111/pbi.70104 (PMC12310839; doi:10.1111/pbi.70104)
Supplement: Supplementary file 2 — Table S2 An overview of infrastructure, equipment and expertise needed for different levels of genomic‐assisted breeding applications for crop improvement programs. [file PBI-23-3119-s002.docx]

**Genomics-assisted breeding for designing salinity-smart future crops**

Ali Raza^1,2^, Qamar U Zaman^3^, Sergey Shabala^4,5^, Mark Tester^6^, Rana Munns^7^, Zhangli Hu^1,2,8*^, Rajeev K. Varshney^9*^

^1^Guangdong Key Laboratory of Plant Epigenetics, College of Life Sciences and Oceanography, Shenzhen University, Shenzhen, China

^2^Shenzhen Engineering Laboratory for Marine Algal Biotechnology, Guangdong Technology Research Center for Marine Algal Biotechnology, Longhua Innovation Institute for Biotechnology, College of Life Sciences and Oceanography, Shenzhen University, Shenzhen, China

^3^School of Breeding and Multiplication, Hainan Yazhou Bay Seed Laboratory, Hainan University, Sanya, China

^4^School of Biological Sciences, The University of Western Australia, Perth, WA, Australia

^5^International Research Centre for Environmental Membrane Biology, Foshan University, Foshan, China

^6^Center of Excellence for Sustainable Food Security and Division of Biological and Environmental Sciences and Engineering, King Abdullah University of Science and Technology (KAUST), Thuwal, Saudi Arabia

^7^Centre of Excellence in Plant Energy Biology, School of Molecular Sciences, The University of Western Australia, Perth, WA, Australia

^8^Guangdong Provincial Key Laboratory of Functional Substances in Medicinal Resources and Healthcare Products, School of Life Sciences and Food Engineering, Hanshan Normal University, Chaozhou, China

^9^WA State Agricultural Biotechnology Centre, Centre for Crop and Food Innovation, Food Futures Institute, Murdoch University, Murdoch, WA, Australia

**^*^Corresponding author**: [huzl@szu.edu.cn](mailto:huzl@szu.edu.cn) / [rajeev.varshney@murdoch.edu.au](mailto:rajeev.varshney@murdoch.edu.au)

**Table S2.** An overview of infrastructure, equipment, and expertise needed for different levels of genomic-assisted breeding applications for crop improvement programs.

| **Breeding levels and GAB tools** | | **Infrastructure and equipment needed** | **Feasibility in developing countries** |
| --- | --- | --- | --- |
| **Basic molecular breeding** | - PCR-based marker selection - Simple genotyping arrays | - PCR machines - Gel electrophoresis setup - Basic DNA extraction kits | - Highly accessible - Minimum cost - Training required |
| Intermediate genomic breeding | - Genomic selection - Genotyping-by-sequencing - Genome-wide association studies - High-throughput phenotyping - Haplotype-based breeding | - Sequencing facilities - Marker-based selection software - Bioinformatics pipelines - Field-based phenotyping platforms | - Partially accessible - Requires moderate funding - Requires moderate expertise |
| Advanced genomic breeding | - Transgenomics - CRISPR - Pan-genomics - AI-driven phenotyping - Epigenomics - Single-cell genomics | - Gene editing labs - High-performance computing - Advanced sequencing platforms - Automated phenotyping systems | - Limited accessible - Requires significant investment - Requires superior expertise |

**Note:** The feasibility of employing genomic-assisted breeding tools will soon vary across regions. In this context, we anticipate that close collaboration between institutions in developed and developing countries, along with capacity-building enterprises, funding support, and technology transfer, can significantly boost accessibility to advanced tools and fast-track crop improvement programs globally without any restrictions.
